# Design, Synthesis, Structural Insights, Tyrosinase Inhibition, and Sun Protection Factor of New Thiosemicarbazone Derivatives

**DOI:** 10.3390/molecules29235629

**Published:** 2024-11-28

**Authors:** Sebastiano Masuri, Benedetta Era, Francesca Pintus, Sonia Floris, Francesca Meloni, Francesca Pettinau, Enrico Podda, Maria Grazia Cabiddu, Antonella Fais, Tiziana Pivetta

**Affiliations:** 1 Department of Chemical and Geological Sciences, University of Cagliari, S.S. 554 Bivio Sestu, Monserrato, 09042 Cagliari, Italy; sebastiano.masuri@unica.it (S.M.); francesca.meloni96t@unica.it (F.M.); francesca.pettinau@unica.it (F.P.); mgcabidd@unica.it (M.G.C.); 2 Department of Life and Environmental Sciences, University of Cagliari, S.S. 554 Bivio Sestu, Monserrato, 09042 Cagliari, Italy; era@unica.it (B.E.); fpintus@unica.it (F.P.); s.floris@unica.it (S.F.); 3 Centre for Research University Services (CeSAR), University of Cagliari, S.S. 554 Bivio Sestu, Monserrato, 09042 Cagliari, Italy; enrico.podda@unica.it

**Keywords:** thiosemicarbazones, tyrosinase, enzyme inhibition

## Abstract

Tyrosinase, a key protein in the biosynthesis of melanin pigments, is crucial in determining skin pigmentation. Inhibiting tyrosinase activity is a promising approach for treating conditions related to excessive pigmentation. For the synthesis of more potent tyrosinase inhibitors, we combined two approaches, para-substitution and lipophilicity, to enhance the inhibitory properties of (*E*)-2-(4-hydroxybenzylidene)hydrazine-1-carbotiamide, whose enzyme inhibitory properties have been previously demonstrated. The newly synthesized compounds showed potent inhibition activity against tyrosinase in the micromolar concentration range. The synthesised compounds were up to 41 times more effective than kojic acid. In addition to this biological activity, all molecules were evaluated for their sun protection factor to determine their photoprotective effects. All the compounds showed higher efficacy than reference compounds, used as sunscreens in photoprotective preparations. All compounds were noncytotoxic at the concentration required to inhibit tyrosinase activity. With the aim of defining the potential binding modes and the kind of interactions between the studied molecules and the catalytic site of mushroom tyrosinase, molecular docking simulations were also performed.

## 1. Introduction

Thiosemicarbazones constitute a class of synthetic organic compounds afforded by the condensation of carbonyl compounds (e.g., aldehydes, ketones) with thiosemicarbazide. These molecules are known for forming stable chelates with different metal ions, thanks to the presence and spatial arrangements of the thiocarbonyl sulphur and hydrazino nitrogen atoms [1,2]. Moreover, many thiosemicarbazones have been evaluated for their anti-tyrosinase properties, observing that these molecules may show potent inhibitory activity towards these macromolecules [3,4,5,6,7,8].

Tyrosinase (EC 1.14.18.1) (Tyr) is the key enzyme of melanogenesis. Tyr contains copper in the active site and performs two sequential enzymatic reactions using molecular oxygen: the ortho-hydroxylation of monophenols and the oxidation of o-diphenols to the corresponding quinones. The reactive quinones then polymerize spontaneously into melanins [9]. Under normal conditions, melanin protects the skin from UV radiation, but its overproduction can cause hyperpigmentation. Different strategies can be exploited to modulate melanogenesis, including inhibiting tyrosinase activity [10,11]. Various methods have been employed to study the Tyr inhibitory activity of samples over the years [12]. Although numerous studies have identified synthetic and natural tyrosinase inhibitors, many of these potential inhibitors are not widely used due to their lack of efficacy and associated toxicities, such as cytotoxicity.

Numerous advances in the biochemical field have allowed a more detailed and precise study of the mechanism of Tyr inhibition [13,14,15]. Optimization of the expression and purification of mushroom and human Tyr has allowed the acquisition of more information into inhibiting these tyrosinases [12]. Depending on the research to be developed, the choice of the appropriate enzyme should be essential. Despite this, most of the studies have been based on in vitro inhibition tests using mushroom Tyr [12,16], which is easily available and cheap, despite the low homology between human and mushroom Tyr and the significant differences in their interaction patterns. However, all Tyr sources share similar copper active sites and it is believed that thiosemicarbazones exert their anti-tyrosinase properties by targeting the very two copper cofactors in the enzyme’s catalytic site [3,4,5]. 

Benzaldehyde-based thiosemicarbazones have proved to possess promising anti-tyrosinase properties. In fact, the introduction of the aromatic ring might increase the affinity between the ligand and the macromolecular target, due to the higher similarity with natural tyrosinase substrates, such as L-Tyrosine and L-dihydroxyphenylalanine (L-DOPA). Moreover, additional hydrophobic interactions with the enzyme’s aminoacidic residues might take place [3]. The anti-tyrosinase activity of benzaldehyde-based thiosemicarbazones seems also to be tuneable according to the position of the substituents in the phenyl ring. For instance, para-substituted derivatives proved to be effective tyrosinase inhibitors, with IC_50_ values ranging in the micromolar order [17,18,19,20]. Compounds **1** (p-Cl), **2** (p-OCH_3_), and **3** (p-NO_2_) are reported as examples in Table 1. 

Lipophilicity also appears to be important in the modulation of anti-tyrosinase activity. Lee et al. observed how the substitution of the phenyl ring (**4**) with a naphthalene one (**5**) increases the inhibitory activity in B16 melanoma cells (Table 1), while the introduction of a more polar ring, like a pyridyl one in compound **6**, determines a loss of the inhibitory activity (IC_50_ > 30 µM). The authors hypothesized that the higher lipophilicity introduced by the naphthalene moiety results in a higher ability of **5** at permeating B16 cell membranes [21]. 

**Figure 1:**
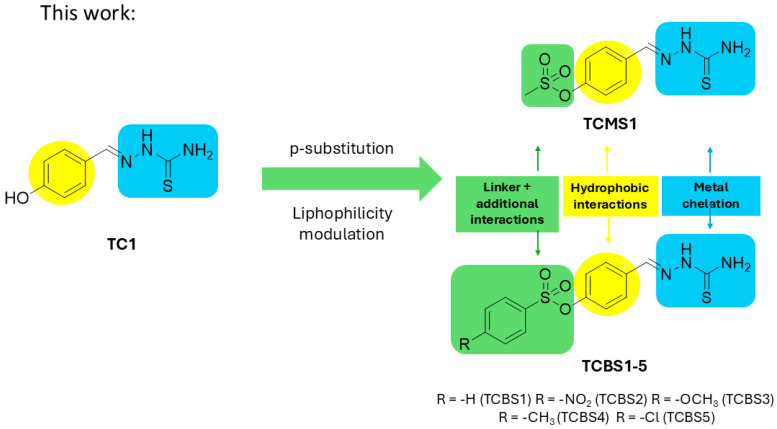
Design strategy and molecular structures of the thiosemicarbazone derivatives proposed in this work.

In this study, we decided to combine the two approaches described above (p-substitution + lipophilicity) with the aim of designing and synthesizing more potent tyrosinase inhibitors. Starting from (E)-2-(4-hydroxybenzylidene)hydrazine-1-carbothioamide (**TC1**, Figure 1), whose enzymatic inhibitory properties were previously reported [20], we functionalized the p-hydroxy group with an alkyl benzenesulfonate moiety (**TCMS1**, Figure 1) and an aryl benzenesulfonate one (compounds **TCBS1-5**, Figure 1). The sulphonate moiety has the main role of linker between the added alkyl/aryl moiety and the pre-existing molecular structure. Additionally, it might also help in stabilizing the adopted bioactive conformation by establishing additional interactions with the surrounding aminoacidic residues of the enzyme. Moreover, we evaluated whether the nature of the substituents could influence the activity of the p-substituted **TCBS2-5** derivatives. 

To give an overall view of the characteristics of the synthesized molecules, several chemical, biological, and theoretical studies were carried out, in particular: X-ray diffraction study, Hirshfeld analysis and molecular descriptors calculations, Tyr inhibition, sun protection factor (SPF), cytotoxicity, antioxidant activity, and copper chelation ability. All these experiments were carried out to obtain useful information on the compound stability and the drug-likeness of the studied molecules, to foresee their behaviour in solution or in polar/apolar media and to ascertain the kind of interaction with the tyrosinase enzyme. All these properties are, in fact, all related to a potential use as drug or in cosmetic formulation.

## 2. Results and Discussion

### 2.1. Synthesis and Chemical Characterisation

**Scheme 1:**
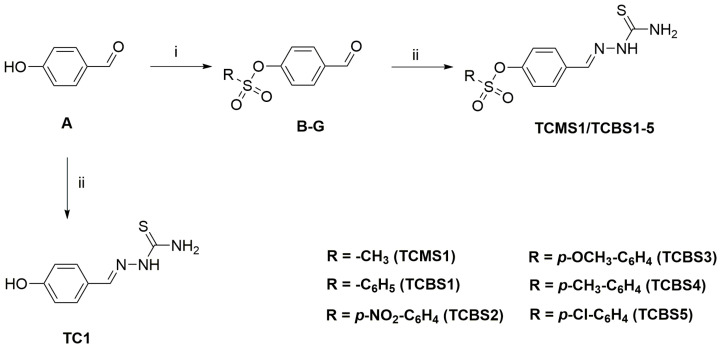
Reaction schemes and structures of the synthesized compounds. Reaction conditions were (i) R−SO_2_Cl, triethylamine, dichloromethane, r.t. overnight; (ii) thiosemicarbazide, absolute ethanol, glacial acetic acid, rf 6 h.

Target compounds **TCMS1** and **TCBS1-5** were easily synthesized (Scheme 1) by reacting 4-hydroxybenzaldehyde (**A**) with the corresponding aryl sulfonyl chlorides, affording the aryl sulfonate intermediates **B**–**G**. These compounds (as crudes) were then converted to the desired thiosemicarbazones in the presence of a catalytic amount of acetic acid. The reference molecule **TC1** was obtained by directly converting **A** to the correspondent thiosemicarbazone derivative, using the synthetic approach just discussed. The yields of the final products ranged from moderate to good. To our knowledge, compounds **TCMS1** and **TCBS1**-**5** have been used as intermediates for the preparation of enzymatic inhibitors (e.g., towards glucosidase, carbonic anhydrase, acetylcholine esterase, butyrylcholine esterase), but their chemical characterization has not been performed [22,23]. For this reason, we characterized the **TCMS1** and **TCBS1-5** molecules employing different techniques (melting point, NMR, HR-ESI-MS). In the ^1^H-NMR spectra of all the reported compounds, characteristic broad singlets from -NH and -NH_2_ protons were visible. Their chemical shift values were in accordance with those reported for **TC1** and other structurally related thiosemicarbazones [19,20,24,25]. The -NH protons were highly de-shielded and fell in the 10.5 ÷ 11.5 ppm range since they were included in a highly conjugated structure. The -NH_2_ protons typically showed up as two separate broad singlets around 8 ppm due to their magnetic inequivalence. This behaviour can be attributed to the partially restricted rotation around the C-N bond due to the mesomeric effect [25], as well as the potential intramolecular hydrogen bonding between one of the NH_2_ hydrogens and the sp2 (imino) nitrogen, which resulted in the formation of a five-membered ring. In order to further prove the nature of these hydrogens, we performed a hydrogen exchange experiment by recording the ^1^H NMR spectrum of **TCBS5** in deuterated acetone before (Figure S12) and after (Figure S14) the addition of D_2_O. As can be seen, addition of deuterium oxide determined a hydrogen/deuterium exchange, which resulted in the disappearance of the aforementioned signals.

Interestingly, the positive HR-ESI-MS spectra of the **TCMS1** and **TCBS1-5** molecules showed a peak at M+17 mass-to-charge ratio (*m*/*z*), corresponding to the protonated S-oxide derivative obtained in the ESI phase. Redox phenomena in positive ESI-MS were already observed for many classes of organic ligands and metal complexes depending on experimental conditions (solvent, needle voltage, housing temperature, etc.) [26,27,28,29,30,31]. The parent ions at M+17 *m*/*z* showed a common loss of 18 Da, corresponding to a water molecule. When the spectra of the same compounds were recorded in negative mode (absence of an oxidizing environment), the analytes did not undergo any redox phenomena and were visible as deprotonated molecular ions ([M−H]^−^) or chlorinated adducts ([M+Cl]^−^). HR-ESI mass spectra in both positive and negative mode for compound **TCMS1** are shown in the Supplementary Material (Figures S15 and S16) as an example. The crystal structure of the **TCBS1** was determined by means of single-crystal X-ray diffraction (SC-XRD). 

### 2.2. Crystal Structure 

**Figure 2:**
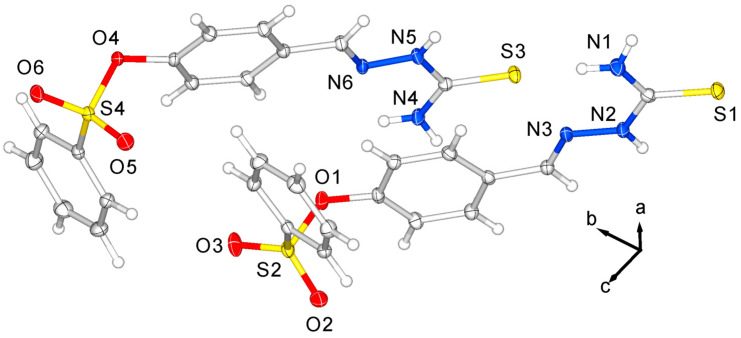
X-ray crystal structure of **TCBS1** showing the heteroatoms’ labelling scheme adopted. Displacement ellipsoids are drawn at the 50% probability level.

Single crystals of **TCBS1** were successfully grown by slow evaporation from chloroform. SC-XRD analysis of a colourless needle-shaped crystal confirmed the nature and chemical connectivity of **TCBS1** that crystallized in the triclinic P1^−^ space group with two crystallographically independent molecules in the asymmetric unit (Figure 2, Table S1).

Thiosemicarbazone moieties in the two molecules of **TCBS1** were coplanar with the plane defined by the vicinal aromatic ring and were in excellent agreement with the geometrical parameters found in this family of structurally characterized derivatives deposited at the CSD (Table S2). On the other hand, the main conformational differences were observed for the dihedral angles C–S–O–C being about 68 and 79° (for C9–S2–O1–C6 and C23–S4–O4–C20, respectively) and for the angle between the planes defined by the two aromatic rings belonging to the same **TCBS1** molecule being about 60 and 82°, respectively. Both molecules of **TCBS1** interacted via N–H∙∙∙S between the sulphur atom and both the amines of the thiosemicarbazone moieties defining a set of R_2^2^ (8) hydrogen-bonding motifs [32] that led to the formation of undulated ribbons developing along the a-axis (Figure S17). The two crystallographically independent molecules of **TCBS1** are depicted as units A (light blue) and B (green) according to Figure S17 and Table S5. The crystal packing was further decorated by slipped π-π stacking interactions between the aromatic rings of the tosyl groups belonging to different units (Figure S18), increasing the dimensionality of the final network (Figure S19).

### 2.3. Hirshfeld Surface

**Figure 3:**
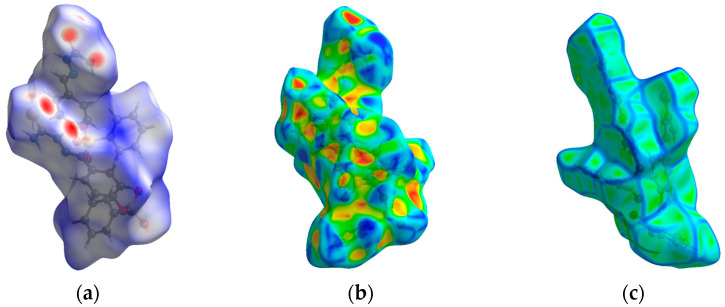
HSs mapped with (**a**) dnorm in the range of −0.4171 a.u. (red) to 1.6374 (blue); (**b**) shape index showing red and blue triangles indicating the π-π stacking areas and (**c**) curvedness with flat areas highlighting the ring contribution in π-π stacking interactions.

**Figure 4:**
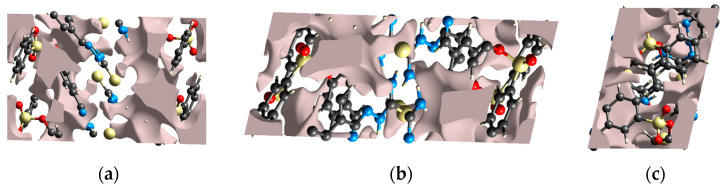
Graphical views of voids in the crystal packing of **TCBS1**, along the projections of [100] (**a**), [010] (**b**), and [001] (**c**). Void volume is 188.45 Å^3^.

Hirshfeld surface (HS) analysis is a modern and powerful tool to analyse the intermolecular interactions present inside the crystal by a simple 3D visualization [33,34,35]. HSs are mapped with respect to four principal parameters, i.e., dnorm (Figure 3a), shape index (Figure 3b), curvedness (Figure 3c), and void (Figure 4). In the dnorm surface, white, red, and blue areas represent contacts close to, shorter than, and longer than the sum of the van der Waals radii, respectively [36,37,38]; the red circles on the surface are indicative of the hydrogen bonding between S1 or S3 sulphur atoms (H acceptors) and the NH groups of neighbouring molecules. The size of the red circle is related to the strength of the interactions, with distance varying from 2.303 Å of S3-HN5 to 2.497 Å of S1-HN1.

The HSs mapped over shape index (Figure 3b) and curvedness (Figure 3b) are useful to describe the effect of weak intermolecular interactions in the crystal. The shape index surface showed complementary depressions indicating the contact areas between two Hirshfeld surfaces [39,40,41,42]. In this map, adjacent red and blue triangular regions are consistent with the presence of π-π stacking: the red triangles are concave zones related to the π-π stacked rings located above, while the blue triangles are convex zones representative of the aromatic rings within the surface. The last map, plotted over the curvedness, depicts the planarity and the sharp edges within the surface. The blue zones are related to the curved surface and large blue areas are indicative of strong hydrogen bonding interactions. The flat zones are due to weak and low-energy interactions [39].

The HS mapped over the void [43,44] shows regions with low electron density. The calculated void volume occupies 12.2% of the total volume of the unit cell, indicating a not rigid crystal packing.

In conclusion, dnorm and curvedness parameters accounted for close and long contacts, π-π stacking, and hydrogen bonding. All these features were involved in the chemical and biochemical reactivity, affecting also the solubility of the compound in different media (polar or apolar). The void percentage is an index of the softness or hardness of the crystal, and this information is useful in the preparation of formulations where the compound could be present in a dispersed phase.

### 2.4. Molecular Descriptors

The preliminary evaluation of the drug-likeness of the studied molecules was performed by means of different molecular descriptors. Results are summarized in Table 2. All the compounds here reported adhered to the Lipinski’s rule of five [45] and showed TPSA values (sum of the surface occupied by polar functional groups) of (i) lower (or almost equal in the case of **TCBS2**) than the upper limit of 140 Å^2^, symptomatic of an oral bioavailability from good to moderate, and (ii) higher than 60 Å^2^, indicative of a modest blood–brain barrier (BBB) permeability [46,47].

### 2.5. Tyrosinase Inhibition

All compounds were evaluated for their inhibitory effect on the tyrosinase enzyme. As can be observed in Table 3, all the tested compounds were found to be significantly more effective than kojic acid. **TC1** had a significantly higher IC_50_ value than other compounds in the series, being only 2.5 times more effective than kojic acid, while the other compounds were up to 41 times more effective than the standard inhibitor.

The results highlighted that the functionalization of the hydroxyl group of **TC1** with a -SO_2_R moiety plays a crucial role in enhancing the anti-tyrosinase activity. Specifically, the -SO_2_R modification appeared to facilitate better binding to the enzyme’s active site. Furthermore, replacing a methyl (**TCMS1**) with a more lipophilic phenyl group in derivatives such as **TCBS1-5** did not significantly reduce the IC_50_ value. The inhibitory activity did not appear to be influenced by the presence and nature of the substituents in the para position of the phenylsulfonyl moiety (**TCBS1-5**). All these considerations suggest that, among the structural modifications made in the studied compounds, introducing sulfonyl groups could be a promising strategy to improve the efficacy of tyrosinase inhibitors.

### 2.6. Antioxidant Activity

Two methods, with DPPH and ABTS, were used to evaluate the antioxidant activity of the synthesized compounds, resulting in similar values for each compound analysed (see Table 4).

As shown in Table 4, the tests highlighted an antioxidant power between 16% and 74% among the compounds examined. Compound **TC1**, which has a hydroxyl group, showed the highest value.

### 2.7. Sun Protection Factor

In addition to all the biological activities examined, all molecules were evaluated for their sun protection factor (SPF) to determine their skin photoprotective effects. Determining the SPF value of compounds with possible skin application could be important since UV rays trigger oxidative stress reactions and progressive skin aging. The SPF values are reported in Table 5. The studied compounds were compared to caffeic acid (CA), ferulic acid (FA), and cinnamic acid (CI), natural components that are used as sunscreens in photoprotective preparations [49], resulting as being more effective.

### 2.8. Copper Chelation Studies

It is well known that Cu(II), as borderline species according to Hard and Soft Acid and Basis Theory (HSAB), can form stable metal complexes with both hard donors (e.g., oxygen) and soft ones (e.g., sulphur) [50]. Many bioactive Cu(II) complexes bearing sulphur-containing functional groups (e.g., thioamides, thiosemicarbazones) or oxygen-based donors (e.g., polyphenols, carboxylates) have been studied at both solution and solid states [51,52,53,54]. Hence, the design of tyrosinase inhibitors having metal chelating groups might be useful thanks to their capability to target the enzyme’s metal cofactors. Based on these premises, we aimed to preliminarily evaluate the capability of the studied molecules to form copper complexes in solution at the same pH used for enzymatic inhibition experiments (6.8), using Job’s method. We selected compound **TCBS4** as a model for the copper chelation study. The Job’s Plots (Figure S20A,B), carried out for this molecule, were obtained from absorbance data recorded at 310 and 318 nm (Figure S20C). The experimental results evidenced the formation of a 1:3 (metal:ligand) complex (χ_L_ = 0.75). Uncorrected absorbance data as a function of ligand’s molar fraction are also shown (Figure S21). It is important to note that these results refer to the metal chelating capabilities of **TCBS4** in a model solution containing Cu^2+^ ions (derived from Cu(II) chloride). In tyrosinase’s catalytic site, the two metal ions were already coordinated by six histidine residues (three for each metal ion). Hence, based on these data, we can assume that the studied molecules might interact with the two metal cofactors by forming mixed complexes with different molar ratios, due to the steric hindrance introduced by the surrounding residues in the enzyme’s catalytic site. This hypothesis seems to be corroborated by molecular theoretical simulations (See Section 2.10).

### 2.9. Cytotoxicity Analysis

**Figure 5:**
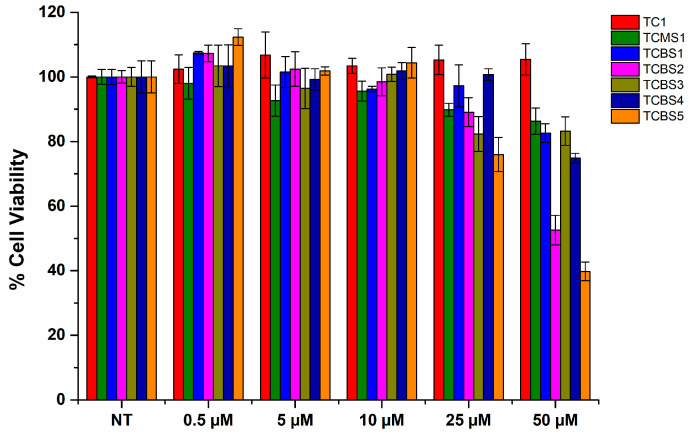
Cell viability of HaCaT cell line after treatment with different concentrations of compounds. Concentrations were from 0 (NT) to 50 µM.

Based on the promising results from earlier experiments, we conducted further evaluations to determine the compounds’ biosafety effectiveness. The results are depicted in Figure 5. Our findings demonstrated that all compounds were noncytotoxic to HaCaT cells at the concentration required to inhibit tyrosinase activity.

### 2.10. Molecular Docking

**Figure 6:**
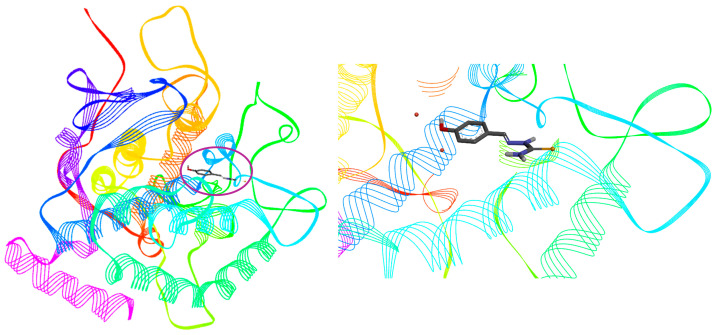
Full (**left**) and zoomed (**right**) view of the highest-rated docked pose of compound **TC1** in the MTa site.

With the aim of assessing the potential binding modes and interactions of the studied molecules in the catalytic site of mushroom tyrosinase (MTa), molecular docking simulations were performed starting from the X-ray structure of the complex (PDB: 2Y9X) between mushroom tyrosinase (from *Agaricus bisporus*) and the natural inhibitor tropolone (OTR). As previously reported [55], the suitability of the computational protocol was verified by redocking the cognate OTR ligand in the enzyme’s binding pocket, affording an acceptable root mean square deviation (RMSD) between the atomic positions of docked and crystallized ligands (2.3 Å). Docking scores and intermolecular interactions are summarized in Table S6. All the studied thiosemicarbazones fit the catalytic site of mushroom tyrosinase, showing two possible binding orientations. In the case of compound **TC1** (Figure 6), its docked pose projected the thiosemicarbazone group towards the enzyme’s outer surface with the p-hydroxyphenyl ring collocated in the enzyme’s binding pocket. This conformation was further stabilized by a hydrogen bond between the ligand’s hydrazido nitrogen and the Gly-281 residue (D-H--A distance: 2.01 Å) and by π-π stacked interactions with the Hys-263 ring (centroid distance: 3.89 Å), as shown in Figure S22.

**Figure 7:**
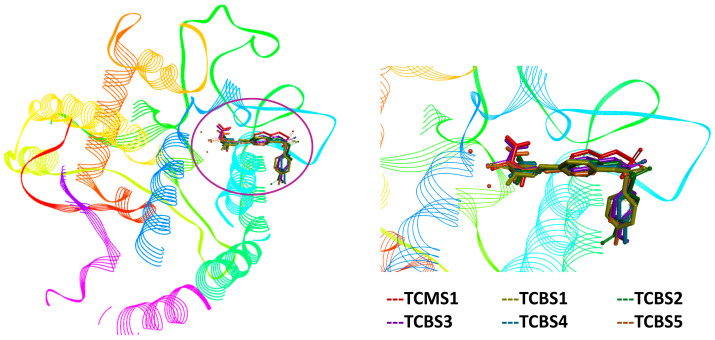
Full (**left**) and zoomed (**right**) view of the highest-rated docked poses of compounds **TCMS1**, **TCBS1-5** in the catalytic site of mushroom tyrosinase.

In the opposite, when the hydroxyl group was functionalized with a -SO_2_R moiety, as in **TCMS1** and **TCBS1-5**, their docked poses underwent a 180° twist, with their thiosemicarbazone groups involved in the coordination of the two copper ions through the ligands’ sulphur atoms (Figure 7 and Figure S23). The conformations of all the thiosemicarbazones were further stabilized by a series of hydrophobic interactions, as observed from a lipophilic analysis of the enzyme’s surrounding residues. In particular, all of them shared hydrophobic interactions between the ligands’ phenyl ring (bonded to the thiosemicarbazone group) and the Val-283 (Figure S24). This residue was involved in stabilizing the bioactive conformation of the tropolone molecule in the 2Y9X complex [55,56,57]. In addition, molecular docking simulations of different inhibitors in the catalytic site of mushroom tyrosinase showed an involvement of the Val-283 residue as well [7,55,57,58,59,60]. Substitution of the methylsulfonyl fragment in **TCMS1** with a more lipophilic phenylsulfonyl one, as in **TCBS1-5**, led to additional hydrophobic interactions between this phenyl ring and the aminoacidic residues Val-248 and Phe-264. These findings might help in explaining the similar anti-tyrosinase potency of the phenyl (**TCBS1-5**) thiosemicarbazone derivatives.

## 3. Materials and Methods

### 3.1. Chemicals

The 4-hydroxybenzaldehyde was purchased from Eastman Organic Chemicals (Kingsport, TN, USA). Absolute ethanol, dichloromethane, sodium sulphate, copper(II) chloride, triethylamine, thiosemicarbazide, p-chlorobenzenesulfonyl chloride, p-toluenesulfonyl chloride, methanesulfonyl chloride, p-nitrobenzenesulfonyl chloride, isopropanol, acetonitrile, methanol, deuterated dimethyl sulfoxide (DMSO d-6), deuterium oxide (D_2_O), deuterated acetone (Acetone d-6), and 2,2′-azino-bis(3-ethylbenzothiazoline-6-sulfonic acid (ABTS) were purchased from Merck (Milan, Italy). The 4-methoxybenzenesulfonyl chloride, sodium dihydrogen phosphate, sodium hydrogen phosphate, and glacial acetic acid were purchased from Thermo Fischer (Kandel, Germany). Benzenesulfonyl chloride was purchased from TCI Europe (Zwijndrecht, Belgium).

### 3.2. Instrumentation Techniques

Melting points were measured on a Kofler Hot Stage (Rochford, UK) and were uncorrected. Proton and Carbon-13 NMR spectra were acquired with a Bruker Advance III HD 600 spectrometer (Rheinstetten, Germany) at room temperature with tetramethylsilane (TMS) as the internal standard in DMSO-d6 or Acetone d-6. Low-resolution ESI mass spectra were acquired with a triple quadrupole (QqQ) Varian 310-MS mass spectrometer (Palo Alto, CA, USA) using previously optimized parameters [61]. High-resolution ESI mass spectra were registered on a Thermofisher ORBITRAP-ELITE instrument (Waltham, MA, USA). The fitting of the isotopic patterns was verified using the mmass 5.5.0 software package [62,63]. UV-vis spectra were recorded using an Agilent Cary 60 spectrophotometer (Palo Alto, CA, USA) with a 1.0 cm quartz cuvette.

### 3.3. General Procedure for the Synthesis of Intermediates B–G

Compounds B–G (Scheme 1) were synthesized by adapting a previously reported method [64]. In brief, to a suspension of *p*-hydroxybenzaldehyde (2.0 mmol, 1 eq) in dichloromethane (8.0 mL) was added at room temperature triethylamine (2.4 mmol, 1.2 eq) followed by the proper sulphonyl chloride derivative (4.0 mmol, 1 eq). The mixture was left under stirring overnight at room temperature, then treated with a saturated aqueous solution of sodium bicarbonate. The organic phase was separated, and the aqueous layer was extracted multiple times with dichloromethane. The combined organic phases were dried over sodium sulphate and evaporated, affording the desired B–G intermediates as solids. The compounds were used as such for the subsequent step, with no further purification procedures.

### 3.4. General Procedure for the Synthesis of Target Compounds ***TC1***, ***TCMS1***, ***TCBS1-5***

The proper aldehyde derivative A-G (1.9 mmoles, 1 eq) was dissolved in ethanol (7.6 mL), then thiosemicarbazide (1.9 mmoles, 1 eq) and glacial acetic acid (0.095 mL) were added. The reaction mixture was refluxed for 6 hrs, then cooled to room temperature, affording a solid that was recovered by filtration. Recrystallization from isopropanol (**TC1**), acetonitrile (**TCMS1**, **TCBS2**), methanol (**TCBS1**), or ethanol (**TCBS3-5**) afforded the desired products as solids.

(*E*)-2-(4-hydroxybenzylidene)hydrazine-1-carbothioamide (**TC1**). Yield: 81%. Experimental results were following those reported in the literature [65], m.p. 221–223 °C; ^1^H NMR (600 MHz, DMSO-d6, Figure S1): δ 11.23 (s, 1H), 9.84 (s, 1H), 8.04 (s, 1H), 7.95 (s, 1H), 7.81 (s, 1H), 7.63–7.58 (m, 2H), 6.80–6.75 (m, 2H); LR-ESI-MS (*m*/*z*) found (calculated): 196.3 (196.0) [M+H]^+^, 217.7 (218.0) [M+Na]^+^.

(*E*)-4-((2-carbamothioylhydrazineylidene)methyl)phenyl methanesulfonate (**TCMS1**). Yield: 78%; m.p. 224–226 °C; ^1^H NMR (600 MHz, DMSO-d6, Figure S2) δ 11.48 (s, 1H), 8.23 (s, 1H), 8.06 (s, 2H), 7.95–7.90 (m, 2H), 7.40–7.34 (m, 2H), 3.40 (s, 3H); ^13^C NMR (151 MHz, DMSO d6, Figure S3) δ 178.1, 149.9, 140.8, 133.4, 129.0, 122.5, 37.5; HR-ESI-MS (*m*/*z*), found (calculated): 272.0141 (272.0164) [M−H]^−^, 290.0285 (290.0269) [M_S-Ox_+H]^+^ (oxidized product).

(*E*)-4-((2-carbamothioylhydrazineylidene)methyl)phenyl benzenesulfonate (**TCBS1**). Yield: 63%; m.p. 159–160 °C; ^1^H NMR (600 MHz, Acetone-d6, Figure S4) δ 10.49 (s, 1H), 8.14 (s, 1H), 7.91 (s, 1H), 7.89–7.87 (m, 2H), 7.84–7.78 (m, 3H), 7.68 (t, *J* = 7.7 Hz, 2H), 7.50 (s, 1H), 7.07 (d, *J* = 8.5 Hz, 2H); ^13^C NMR (151 MHz, Acetone d6, Figure S5) δ δ 180.7, 151.4, 141.7, 136.1, 135.6, 134.5, 130.5, 129.5, 129.3, 123.5; HR-ESI-MS (*m*/*z*), found (calculated): 334.0305 (334.0320) [M−H]^−^, 370.0066 (370.0087) [M+Cl]^−^ with the expected isotopic pattern, 352.0450 (352.0426) [M_S-Ox_+H]^+^ (oxidized product).

(*E*)-4-((2-carbamothioylhydrazineylidene)methyl)phenyl 4-nitrolbenzenesulfonate (**TCBS2**). Yield: 61%; m.p. 226–228 °C; ^1^H NMR (600 MHz, DMSO-d6, Figure S6) δ 11.47 (s, 1H), 8.48–8.43 (m, 2H), 8.23 (s, 1H), 8.21–8.14 (m, 2H), 8.04 (s, 1H), 8.00 (s, 1H), 7.87–7.82 (m, 2H), 7.13–7.08 (m, 2H); ^13^C NMR (151 MHz, DMSO d6, Figure S7) δ 178.2, 151.1, 149.3, 140.4, 139.4, 133.9, 130.1, 129.0, 125.0, 122.4; HR-ESI-MS (*m*/*z*), found (calculated): 379.0145 (379.0171) [M−H]^−^, 414.9907 (414.9938) [M+Cl]^−^ with the expected isotopic pattern, 397.0312 (397.0277) [M_S-Ox_+H]^+^ (oxidized product).

(*E*)-4-((2-carbamothioylhydrazineylidene)methyl)phenyl 4-methoxylbenzenesulfonate (**TCBS3**). Yield: 48%; m.p. 154–156 °C; ^1^H NMR (600 MHz, Acetone-d6, Figure S8) δ 10.50 (s, 1H), 8.14 (s, 1H), 7.91 (s, 1H), 7.82–7.76 (m, 4H), 7.50 (s, 1H), 7.18–7.13 (m, 2H), 7.08–7.03 (m, 2H), 3.93 (s, 3H); ^13^C NMR (151 MHz, Acetone d6, Figure S9) δ δ 180.7, 165.4, 151.6, 141.8, 134.3, 131.7, 129.5, 127.3, 123.6, 115.6, 56.4; HR-ESI-MS (*m*/*z*), found (calculated): 364.0406 (364.0426) [M−H]^−^, 400.0162 (400.0193) [M+Cl]^−^ with the expected isotopic pattern, 382.0562 (382.0531) [M_S-Ox_+H]^+^ (oxidized product).

(*E*)-4-((2-carbamothioylhydrazineylidene)methyl)phenyl 4-methylbenzenesulfonate (**TCBS4**). Yield: 76%; m.p. 195–197 °C; ^1^H NMR (600 MHz, Acetone-d6, Figure S10) δ 10.49 (s, 1H), 8.13 (s, 1H), 7.90 (s, 1H), 7.83–7.77 (m, 2H), 7.76–7.72 (m, 2H), 7.52–7.46 (m, 3H), 7.09–7.04 (m, 2H), 2.46 (s, 3H); ^13^C NMR (151 MHz, Acetone d6, Figure S11) δ 180.7, 151.5, 146.9, 141.8, 134.4, 133.2, 130.9, 129.5, 129.3, 123.5, 21.6; HR-ESI-MS (*m*/*z*), found (calculated): 348.0454 (348.0477) [M−H]^−^, 384.0214 (384.0243) [M+Cl]^−^ with the expected isotopic pattern, 366.0608 (366.0582) [M_S-Ox_+H]^+^ (oxidized product).

(*E*)-4-((2-carbamothioylhydrazineylidene)methyl)phenyl 4-chlorobenzenesulfonate (**TCBS5**). Yield: 76%; m.p. 194–196 °C; ^1^H NMR (600 MHz, Acetone-d6, Figure S12) δ 10.47 (s, 1H), 8.14 (s, 1H), 7.92–7.86 (m, 3H), 7.85–7.80 (m, 2H), 7.75–7.70 (m, 2H), 7.49 (s, 1H), 7.13–7.07 (m, 2H); ^13^C NMR (151 MHz, Acetone d6, Figure S13) δ 180.7, 151.2, 141.7, 141.6, 134.7, 134.6, 131.1, 130.7, 129.6, 123.5; HR-ESI-MS (*m*/*z*), found (calculated): 367.9898 (367.9930) [M−H]^−^ with the expected isotopic pattern, 403.9659 (403.9697) [M+Cl]^−^ with the expected isotopic pattern, 386.0067 (386.0036) [M_S-Ox_+H]^+^ (oxidized product) with the expected isotopic pattern.

### 3.5. Crystal Structure

Single-crystal X-ray diffraction data of **TCBS1** were collected at 100 K on a Bruker D8 Venture diffractometer equipped with a PHOTON II detector. The structure was solved with the ShelXT [66] solution program using dual methods and developed by iterative cycles of least-squares refinement on F2 using ShelXL 2018/3 [66]. Olex2 1.5 [67] was used as the graphical interface and for the preparation of figures. Hydrogen atoms were placed geometrically and refined isotropically riding on their parent C atom with Uiso(H) = 1.2Ueq(C). H atoms bonded to heteroatoms were located from the difference Fourier map and their positions were refined freely. Crystallographic data were deposited at the Cambridge Crystallographic Data Center (CCDC) under deposition number: 2374503. These data can be obtained free of charge at: https://www.ccdc.cam.ac.uk/structures.

### 3.6. Hirshfeld Analysis

Hirshfeld analysis was carried out on structural data with Crystal Explorer software (v. 21.5, Rev. 608bb.32) [32].

### 3.7. Molecular Descriptors

All the calculations were performed using the Molinspiration property engine (v2022.08, accessed on 16 May 2024).

### 3.8. Tyrosinase Inhibition Assay

Mushroom Tyr inhibition was assessed as described previously [11] with minor modifications. A solution consisting of 50 mM of phosphoric acid buffer (pH 6.8), mushroom tyrosinase solution (Sigma Chemical Co., Milan, Italy) at a final concentration of 72 U/mL, and DMSO or the compounds tested was incubated at 37 °C for 10 min; L-DOPA was then added and the dopachrome formation was monitored at 492 nm. The measurements were taken using a FLUOstar OPTIMA (BMG Labtech, Offenburg, Germany). The IC_50_ value was determined by analysing dose–response curves. Kojic acid was used as a reference tyrosinase inhibitor.

### 3.9. Antioxidant Assays

#### 3.9.1. DPPH

Radical scavenging activity of the thiosemicarbazone derivatives was estimated according to the previously reported method [68] with slight modification using the stable DPPH radical. A solution of 0.1 mM DPPH radical was added to various concentrations of the compounds. The absorbance of the DPPH radical without an antioxidant, i.e., blank, was also measured. The mixture was shaken vigorously and kept at room temperature for 30 min in the dark. The absorbance of the reaction mixture was measured at 517 nm spectrophotometrically. All the determinations were performed in triplicate.

#### 3.9.2. ABTS

Samples of each compound (10 µL) were added to 990 µL of ABTS, and the reduction in the blue-green radical ABTS^+•^ by hydrogen-donating antioxidants was evaluated by measuring the absorbance at 734 nm after 1 min of incubation. All the measurements were carried out at least three times.

### 3.10. Sun Protection Factor (SPF)

The sun protection factor of thiosemicarbazone derivatives was measured by the UV absorbance method, as previously reported [11]. The absorbances of the compounds (50 µM) were recorded in the range of 290–320 nm, with 5 nm increments, and three measurements were performed at each point. The SPF was calculated by using the Mansur Equation:

$$SPF=CF\times \sum_{320}^{290}EE(\lambda )\times I(\lambda )\times Abs(\lambda )$$

where CF = correction factor (10); EE (λ) = erythemogenic effect of radiation with wavelength λ; I(λ)  = solar intensity spectrum; and Abs(λ) = spectrophotometric absorbance values at wavelength λ. The values of EE (λ)×I(λ)  are constant, as determined by Sayre et al. [69].

### 3.11. Copper Chelation Studies

Stock solutions of **TCBS4** and Copper(II) chloride dihydrate 3.20 mM were prepared in DMSO, then diluted to 32 µM in 50 mM Phosphate buffer pH 6.8 (percentage of DMSO in the diluted solutions ≈ 1%). Eleven solutions having a variable molar ratio of the two components (from 0:10 to 10:0) but fixed final volume (2.0 mL) and total molar concentration (32 µM) were prepared, and the UV-vis spectra were recorded in the 250–500 nm range. Absorbance data were corrected from the contribution of the pure reactants.

### 3.12. Cell Viability Assay

The cellular cytotoxicity of compounds was investigated using a 3-(4,5-dimethylthiazol-2-yl)-2,5-diphenyl-tetrazolium bromide (MTT) assay [70]. The HaCaT cell line of human keratinocytes was obtained from CLS-Cell Line Services in Eppelheim, Germany. The cells were exposed for 24 h to compounds at concentrations ranging from 0.5 to 50 µM. Then, MTT reagent (0.5 mg/mL in DMEM) was added to each well. The plate was incubated for 3 h at 37 °C. The MTT solution was removed from the culture plate, and 100 μL of DMSO solvent was added to solubilize the water-insoluble formazan crystals formed in the cells. The absorbance was determined at 570 nm using a microplate reader (VANTAstar_BMG LABTECH GmbH, Offenburg, Germany).

### 3.13. Docking Calculations

Molecular docking simulations were performed using the CCDC GOLD software (v2024.1.0, Cambridge, UK) [71]. The crystal structure of the adduct formed between mushroom tyrosinase (from *Agaricus bisporus*) and the tropolone (OTR) inhibitor (PDB code: 2Y9X) was chosen as receptor [56]. Equilibrium conformer of each molecule was assessed at molecular mechanics level and optimized at PM3 level [72,73] using Spartan v.2014. The resulting structures was then optimized at the Density Functional Theory (DFT) level with Orca 5.0.4 [74,75] (PBE0 density functional [76], def2-SVP basis set [77]). The nature of the minima achieved after the optimization procedures was confirmed by the absence of negative IR frequencies in the Hessian matrix. The computational setup adopted for the molecular docking simulations was already described and validated [55]. The conformations adopted by the docked poses and their intermolecular interactions were analysed using Biovia Discovery studio viewer 2021, USCF Chimera v. 1.8 [78] and USCF ChimeraX v. 1.8 [79]. Analysis of the hydrophobic interactions was performed by colouring the surrounding residues according to the Kyte–Doolittle hydrophobicity scale values [80]. 

### 3.14. Statistical Analysis

Statistically significant differences were assessed by calculating a one-way ANOVA followed by the Tukey Multiple Comparisons Test, both using the Graph Pad INSTAT software v8.2 (GraphPad Software, San Diego, CA, USA).

## 4. Conclusions

In this work, we have reported the design and synthesis of a new series of tyrosinase inhibitors bearing the thiosemicarbazones fragment, by introducing the combination of para-substitution and lipophilicity-exploiting strategies. This approach enabled the development of compounds with inhibitory effects on tyrosinase significantly higher than those shown by the standard inhibitor kojic acid, highlighting their potential for treating hyperpigmentation and related skin problems. With a view to the possible dermatological use, it is worthy to remark that the synthesized thiosemicarbazones show promising photoprotective properties, with SPF values higher than those of the commonly used sunscreen agents. Importantly, cytotoxicity experiments verified the non-toxicity of our compounds at concentrations efficient for tyrosinase inhibition. 

Molecular docking experiments revealed useful information about these compounds’ binding interactions with the active site of mushroom tyrosinase, clarifying their mechanism of action as possible anti-tyrosinase drugs. The capability of selected compounds to chelate critical copper ions in the enzyme increases their inhibitory potency and applications.

In conclusion, the thiosemicarbazone derivatives synthesized in this study represent a promising class of compounds for potential therapeutic and cosmetic applications aimed at regulating melanin production and providing UV protection. The results obtained open promising perspectives for further in vitro studies with human tyrosinase and on cellular systems for melanogenesis studies.

## Figures and Tables

**Table 1:** Tyrosinase inhibitory activity (using L-DOPA as substrate), expressed as IC_50_, for p-substituted **1**–**3** thiosemicarbazones and anti-melanogenesis activity (on B16 melanoma cells), reported as IC50, expressed as a function of lipophilicity for **4**–**6** thiosemicarbazones.

Molecule	Tyrosinase Inhibitory Activity IC_50_ (µM)	Anti-Melanogenesis Activity IC_50_ (µM)	cLogP	References
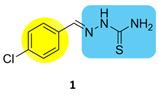	0.84 1.9	-	-	[17,18]
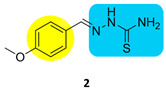	0.44 1.48	-	-	[17,20]
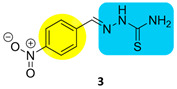	1.9	-	-	[19]
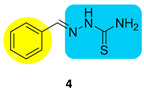	-	>10	1.865	[21]
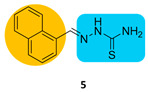	-	1.1	3.039	[21]
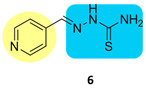	-	>30	1.088	[21]

**Table 2:** Calculated molecular descriptors for the studied compounds.

	TC1	TCMS1	TCBS1	TCBS2	TCBS3	TCBS4	TCBS5
miLogP ^a^	1.40	1.31	2.85	2.81	2.90	3.30	3.52
TPSA (Å^2^) ^b^	70.64	93.79	93.79	139.61	103.02	93.79	93.79
n-atoms ^c^	13	17	22	25	24	23	23
MW (Da)	195.25	273.34	335.41	380.41	365.44	349.44	369.86
n-ON ^d^	4	5	5	7	6	5	5
n-OHNH ^e^	4	3	3	3	3	3	3
n-violations ^f^	0	0	0	0	0	0	0
n-rotb ^g^	3	5	6	7	7	6	6
volume (Å^3^) ^h^	166.87	215.83	270.68	294.01	296.23	287.24	284.22

^a^ Calculated logarithm of the partition coefficient between n-octanol and water (miLogP); ^b^ topological polar surface area (TPSA); ^c^ number of atoms in the molecule (n-atoms); ^d^ number of hydrogen bond acceptors (n-ON); ^e^ number of hydrogen bond donors (n-OHNH); ^f^ number of violations of the Lipinski’s rule of five; ^g^ number of rotatable bonds (n-rotb); ^h^ molecular volume.

**Table 3:** Half maximal inhibitory concentration (IC_50_) values of **TC1**, **TCMS1**, and **TCBS1-5** compounds against mushroom tyrosinase activity (data are given as mean ± standard deviation (SD) of triplicate experiments).

Compound	IC_50_ µM *
**TC1**	7.1 ± 0.2 ^a^
**TCMS1**	1.6 ± 0.02 ^b^
**TCBS1**	0.44 ± 0.03 ^b^
**TCBS2**	0.66 ± 0.05 ^b^
**TCBS3**	0.44 ± 0.01 ^b^
**TCBS4**	0.44 ± 0.01 ^b^
**TCBS5**	0.71 ± 0.02 ^b^
**Kojic acid**	18 ± 1 ^c^ [48]

* Different letters indicate statistically significant differences between compounds (*p* < 0.001).

**Table 4:** Free radical scavenging activity (**TC1**, **TCMS1**, and **TCSB1-5**) as a percentage of inhibition of free radicals (I). The data of triplicate experiments are given as mean ± standard deviation (SD).

Compound	DPPH (% I ± SD)	ABTS (% I ± SD)
**TC1**	74 ± 3	74 ± 1
**TCMS1**	20 ± 2	42 ± 4
**TCBS1**	26 ± 2	39 ± 1
**TCBS2**	16 ± 2	25 ± 4
**TCBS3**	33 ± 4	35 ± 1
**TCBS4**	20 ± 1	30 ± 2
**TCBS5**	32 ± 5	22 ± 2

**Table 5:** Sun protection factor (SPF) values of thiosemicarbazone derivates (**TC1**, **TCMS1**, **TCBS1**-**5** compounds) and ferulic acid (**FA**), caffeic acid (**CA**), and cinnamic acid (**CI**).

Compound	SPF *
**TC1**	11.0 ± 0.4 ^a,b^
**TCMS1**	11 ± 2 ^a,b^
**TCBS1**	11 ± 2 ^a,b^
**TCBS2**	13 ± 2 ^a^
**TCBS3**	10 ± 2 ^a,b^
**TCBS4**	14 ± 2 ^a^
**TCBS5**	11 ± 2 ^a,b^
**FA ^#^**	7.5 ± 0.2 ^b^
**CA ^#^**	6.6 ± 0.4 ^b^
**CI ^#^**	2.0 ± 0.2 ^d^

* Different letters indicate statistically significant differences between compounds (*p* < 0.001). ^#^ Data taken from reference [49].

## Data Availability

Data are present within the article.

## Supplementary Materials

The following supporting information can be downloaded at https://www.mdpi.com/article/10.3390/molecules29235629/s1: Figure S1: ^1^H NMR spectrum of **TC1**, (*E*)-2-(4-hydroxybenzylidene)hydrazine-1-carbothioamide (600 MHz, DMSO d-6); Figure S2: ^1^H NMR spectrum of **TCMS1**, (*E*)-4-((2-carbamothioylhydrazineylidene)methyl)phenyl methanesulfonate (600 MHz, DMSO d-6); Figure S3: ^13^C NMR spectrum of **TCMS1**, (*E*)-4-((2-carbamothioylhydrazineylidene)methyl)phenyl methanesulfonate (151 MHz, DMSO d-6); Figure S4: ^1^H NMR spectrum of **TCBS1**, (*E*)-4-((2-carbamothioylhydrazineylidene)methyl)phenyl benzenesulfonate (600 MHz, Acetone-d6); Figure S5: ^13^C NMR spectrum of **TCBS1**, (*E*)-4-((2-carbamothioylhydrazineylidene)methyl)phenyl benzenesulfonate (151 MHz, Acetone-d6); Figure S6: ^1^H NMR spectrum of **TCBS2**, (*E*)-4-((2-carbamothioylhydrazineylidene)methyl)phenyl 4-nitrobenzenesulfonate (600 MHz, DMSO d-6); Figure S7: ^13^C NMR spectrum of **TCBS2**, (*E*)-4-((2-carbamothioylhydrazineylidene)methyl)phenyl 4-nitrobenzenesulfonate (151 MHz, DMSO d-6); Figure S8: ^1^H NMR spectrum of **TCBS3**, (*E*)-4-((2-carbamothioylhydrazineylidene)methyl)phenyl 4-methoxybenzenesulfonate (600 MHz, Acetone d-6); Figure S9: ^13^C NMR spectrum of **TCBS3**, (*E*)-4-((2-carbamothioylhydrazineylidene)methyl)phenyl 4-methoxybenzenesulfonate (151 MHz, Acetone d-6); Figure S10: ^1^H NMR spectrum of **TCBS4**, (*E*)-4-((2-carbamothioylhydrazineylidene)methyl)phenyl 4-methylbenzenesulfonate (600 MHz, Acetone d-6); Figure S11: ^13^C NMR spectrum of **TCBS4**, (*E*)-4-((2-carbamothioylhydrazineylidene)methyl)phenyl 4-methylbenzenesulfonate (151 MHz, Acetone d-6); Figure S12: ^1^H NMR spectrum of **TCBS5**, (*E*)-4-((2-carbamothioylhydrazineylidene)methyl)phenyl 4-chlorobenzenesulfonate (600 MHz, Acetone d-6); Figure S13: ^13^C NMR spectrum of **TCBS5**, (*E*)-4-((2-carbamothioylhydrazineylidene)methyl)phenyl 4-chlorobenzenesulfonate (151 MHz, Acetone d-6); Figure S14: ^1^H NMR spectrum of **TCBS5**, (*E*)-4-((2-carbamothioylhydrazineylidene)methyl)phenyl 4-chlorobenzenesulfonate after addition of D_2_O (600 MHz, Acetone d-6); Figure S15: High-Resolution ESI mass spectrum (positive mode) of compound **TCMS1**; Figure S16: High-Resolution ESI mass spectrum (negative mode) of compound **TCMS1**; Table S1: Crystal data and structure refinement parameters for **TCSB1**; Table S2: Bond lengths (Å) for compound **TCSB1**; Table S3: Bond angles (°) for compound **TCSB1**; Table S4: Comparison between the mean bond distances (Å) calculated for the thiosemicarbazone moieties in the two units in **TCBS1** and the mean values retrieved from the CSD (version 5.45 updated Mar 2024); Figure S17: Hydrogen bonding network found in the crystal structure of **TCBS1**: (a) partial view on the relative orientation between units A and B. (b,c) show the infinite hydrogen-bonded network for units A and B, respectively. Interactions are labelled according to Table S5; Table S5: Intermolecular hydrogen bonding interactions of **TCBS1**; Figure S18: Intermolecular π-π stacking interactions between tosyl groups in the crystal structure of **TCBS1**. Intercentroid distance: 3.90 Å; shift distance: 1.46 Å; plane to plane angle: 9°; Figure S19: Partial view of the packing diagrams of **TCBS1** along the *a*- (left) and *b*-axis (right). Units A and B are depicted in light blue and green, respectively; Figure S20: Job’s plot of Cu^2+^ and **TCBS4** at 310 nm (**A**) and 318 (**B**); (**C**) Absorption spectra collected by varying Cu^2+^and **TCBS4** molar ratios in in PB 0.05 M, pH 6.8, 25 °C, 1 cm optical path length; Figure S21: Uncorrected absorbance data at 310 nm (**A**) and 318 nm (**B**) recorded by varying Cu^2+^and **TCBS4** molar ratios; Figure S22: Docked pose of **TC1** in the MTa site and intermolecular interactions with the surrounding residues. Hydrogen bonds are represented using light blue solid lines, while metal coordinating bonds are shown as violet dashed ones (**A**). π-π interaction between the docked pose of **TC1** and the Hys-263 residue; Figure S23: Docked poses of **TCMS1** (**A**), **TCBS1-5** (**B-F)** in the MTa site and intermolecular interactions with the surrounding residues. Hydrogen bonds are represented using light blue solid lines, while metal coordinating bonds are shown as violet dashed ones; Figure S24: Hydrophobic interaction analysis between the docked poses of **TC1** (**A**), **TCMS1** (**B**), **TCBS1-5** (**C-G**) and the surrounding residues of mushroom tyrosinase (MTa site). The aminoacidic residues are coloured according to the Kyte-Doolittle hydrophobicity scale values; Table S6: Scores, molecular interactions and distances between the highest-rated docked poses of compounds **TC1**, **TCMS1**, **TCBS1-5** and the surrounding residues of mushroom tyrosinase (MTa).
