# Supplementary material for: Design, Synthesis, Structural Insights, Tyrosinase Inhibition, and Sun Protection Factor of New Thiosemicarbazone Derivatives
Source: Molecules. 2024 Nov 28;29(23):5629. doi: 10.3390/molecules29235629 (PMC11643516; doi:10.3390/molecules29235629)
Supplement: Supplementary file 1 [file molecules-29-05629-s001.zip › molecules-3308435-supplementary.pdf]

Supporting Information

Design, Synthesis, Structural Insights, Tyrosinase Inhibition, and Sun Protection Factor of New Thiosemicarbazone Derivatives.

Sebastiano Masuri[1,†], Benedetta Era[2,†], Francesca Pintus[2], Sonia Floris[2], Francesca Meloni[1], Francesca Pettinau[1], Enrico Podda[3], Maria Grazia Cabiddu[1], Antonella Fais[2,*], Tiziana Pivetta[1,*]

[1] Department of Chemical and Geological Sciences, University of Cagliari, S.S. 554 Bivio Sestu, Monserrato, 09042 Cagliari, Italy.

[2] Department of Life and Environmental Sciences, University of Cagliari, .S. 554 Bivio Sestu, Monserrato, 09042 Cagliari, Italy.

[3] Centre for Research University Services (CeSAR), University of Cagliari, S.S. 554 Bivio Sestu, Monserrato, 09042 Cagliari, Italy.

* Correspondence: fais@unica.it (A.F.), tpivetta@unica.it (T.P.).

† These authors contributed equally to this work

Table of Contents

1. Synthesis and chemical characterization

1.1. ^{1}H NMR spectrum of **TC1** **Figure S1**

1.2. ^{1}H NMR spectrum of **TCMS1** **Figure S2**

1.3. ^{13}C NMR spectrum of **TCMS1** **Figure S3**

1.4. ^{1}H NMR spectrum of **TCBS1** **Figure S4**

1.5. ^{13}C NMR spectrum of **TCBS1** **Figure S5**

1.6. ^{1}H NMR spectrum of **TCBS2** **Figure S6**

1.7. ^{13}C NMR spectrum of **TCBS2** **Figure S7**

1.8. ^{1}H NMR spectrum of **TCBS3** **Figure S8**

1.9. ^{13}C NMR spectrum of **TCBS3** **Figure S9**

1.10. ^{1}H NMR spectrum of **TCBS4** **Figure S10**

1.11. ^{13}C NMR spectrum of **TCBS4** **Figure S11**

1.12. ^{1}H NMR spectrum of **TCBS5** **Figure S12**

1.13. ^{13}C NMR spectrum of **TCBS5** **Figure S13**

1.14 ^{1}H NMR spectrum of **TCBS5** after addition of D_2O **Figure S14**

1.14. High-Resolution ESI mass spectrum (positive mode) of **TCMS1** **Figure S15**

1.15. High-Resolution ESI mass spectrum (negative mode) of **TCMS1** **Figure S16**

2. Structural characterization

2.1 Crystal data and structure refinement parameters for **TCSB1**. **Table S1**

2.2 Bond lengths (Å) for compound **TCSB1** **Table S2**

2.3 Bond angles (°) for compound **TCSB1** **Table S3**

2.4 Comparison between the mean bond distances (Å) calculated for the thiosemicarbazone moieties in the two units in **TCBS1** and the mean values retrieved from the CSD **Table S4**

2.5 Hydrogen bonding network found in the crystal structure of **TCBS1** **Figure S17**

2.6 Intermolecular hydrogen bonding interactions of **TCBS1** **Table S5**

2.7 Intermolecular π-π stacking interactions in the crystal structure of **TCBS1** **Figure S18**

2.8 Partial view of the packing diagrams of **TCBS1** along the *a*- and *b*-axis **Figure S19**

3. Copper Chelation studies

3.1. Absorbance spectra and Job's Plots of the **TCBS4**-Cu^{2+} system **Figure S20**

3.2. Uncorrected absorbances at 310 and 318 nm of the **TCBS4**-Cu^{2+} system **Figure S21**

4. Molecular Docking

4.1. Intermolecular interaction profile of **TC1** in the MTa site **Figure S22**

4.2. Intermolecular interaction profile of **TCMS1**, **TCBS1-5** in the MTa site **Figure S23**

4.3. Hydrophobic interactions between the ligands and the surrounding residues **Figure S24**

4.4. Scores, interaction types and distances between the docked ligands and the enzyme's surrounding residues **Table S6**

1. Synthesis and chemical characterization

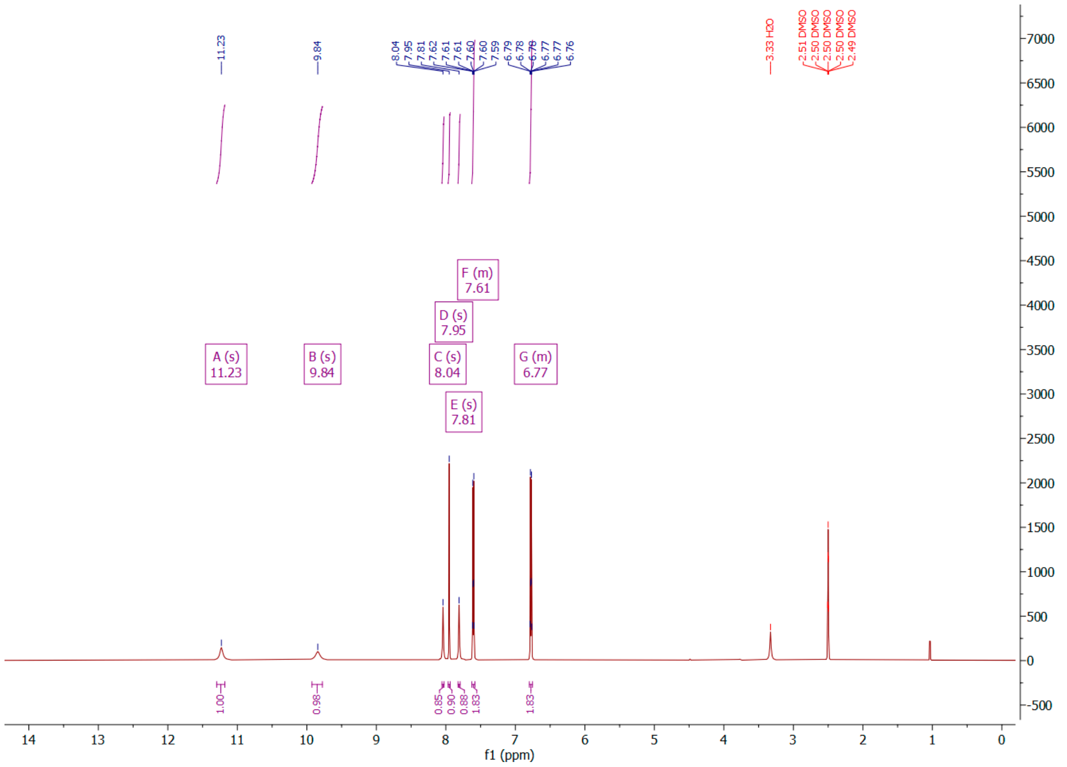

Figure S1. ^{1}H NMR spectrum of **TC1**, (*E*)-2-(4-hydroxybenzylidene)hydrazine-1-carbothioamide (600 MHz, DMSO d-6).

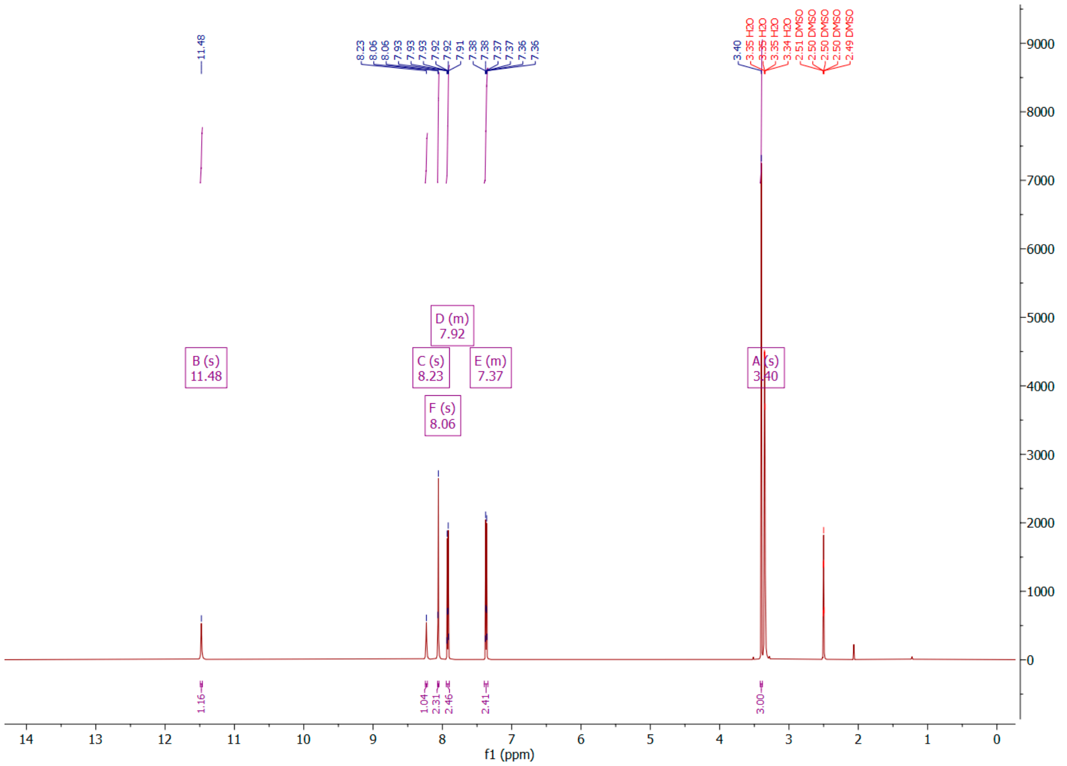

Figure S2. ^{1}H NMR spectrum of **TCMS1**, (*E*)-4-((2-carbamothioylhydrazineylidene)methyl)phenyl methanesulfonate (600 MHz, DMSO d-6).

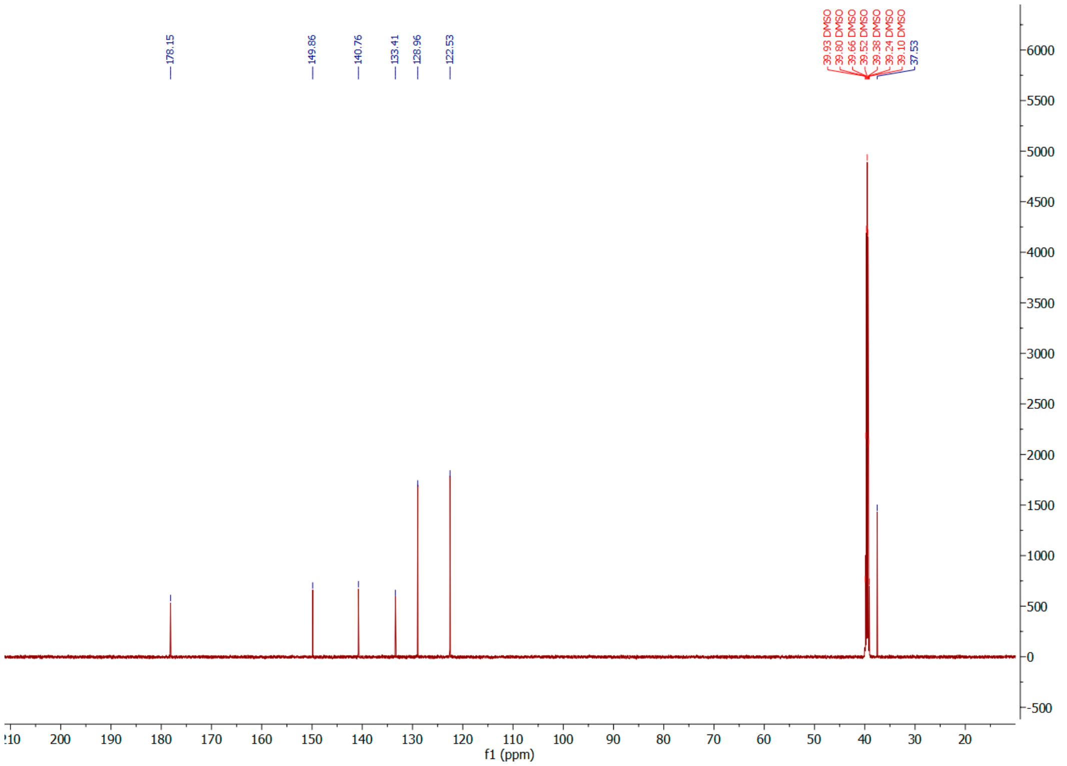

Figure S3. ^{13}C NMR spectrum of **TCMS1**, (*E*)-4-((2-carbamothioylhydrazineylidene)methyl)phenyl methanesulfonate (151 MHz, DMSO d-6).

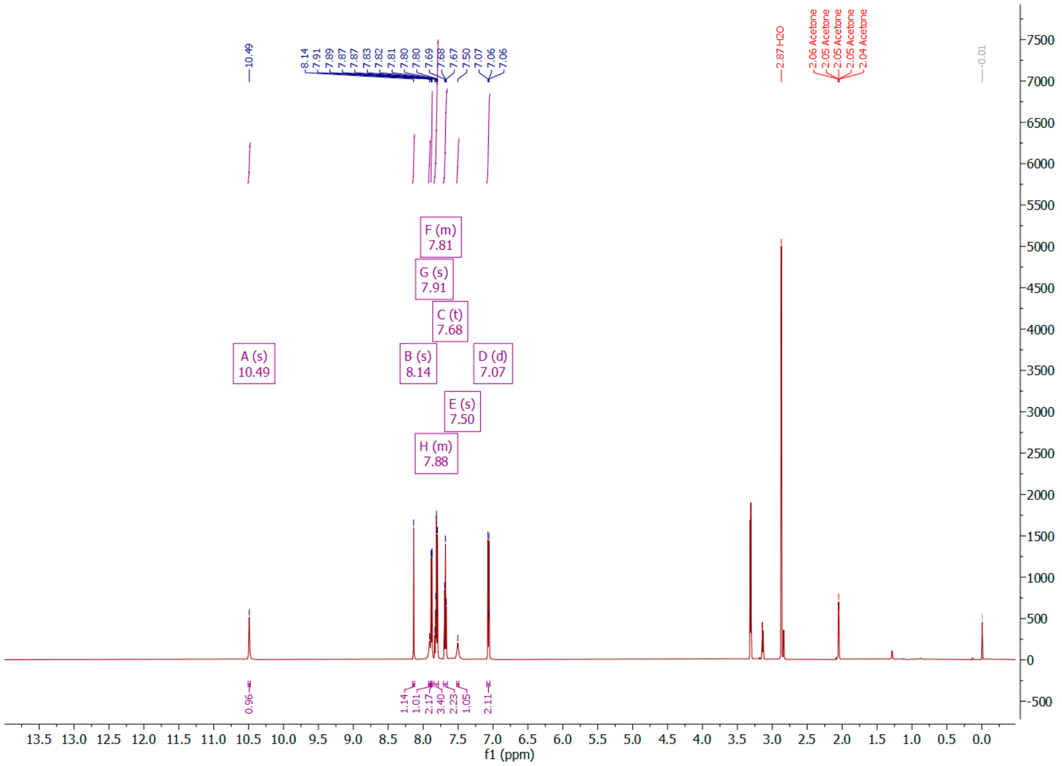

Figure S4. ^{1}H NMR spectrum of **TCBS1**, (*E*)-4-((2-carbamothioylhydrazineylidene)methyl)phenyl benzenesulfonate (600 MHz, Acetone-d6).

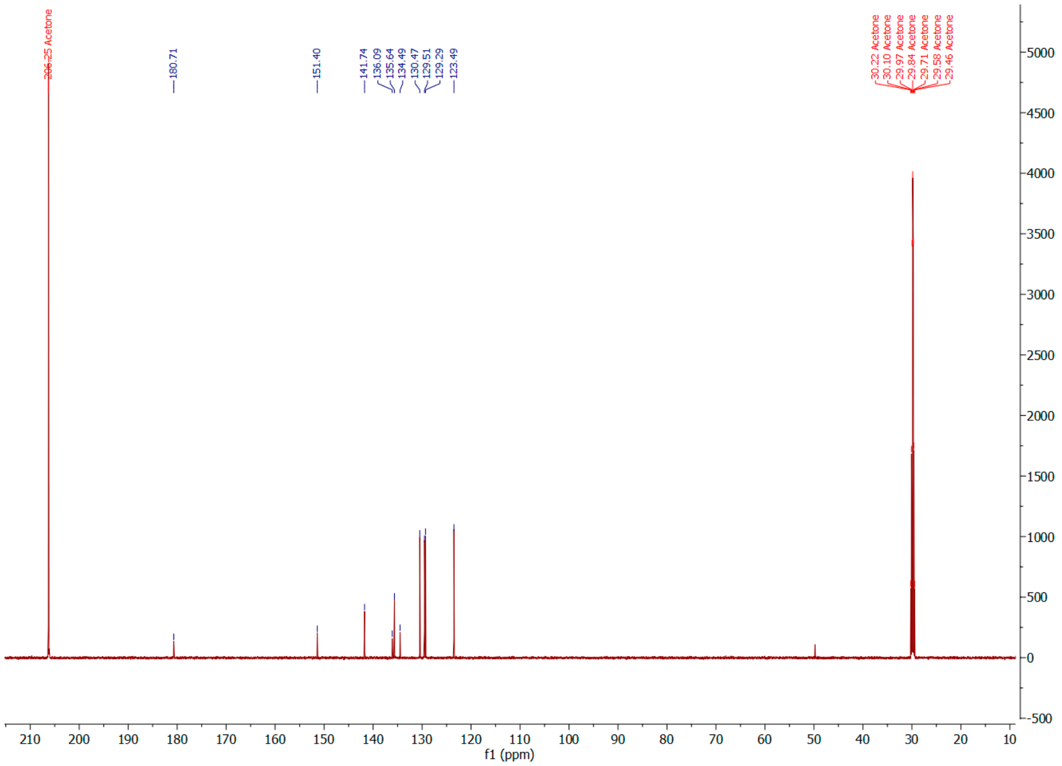

Figure S5. ^{13}C NMR spectrum of **TCBS1**, (*E*)-4-((2-carbamothioylhydrazineylidene)methyl)phenyl benzenesulfonate (151 MHz, Acetone-d6).

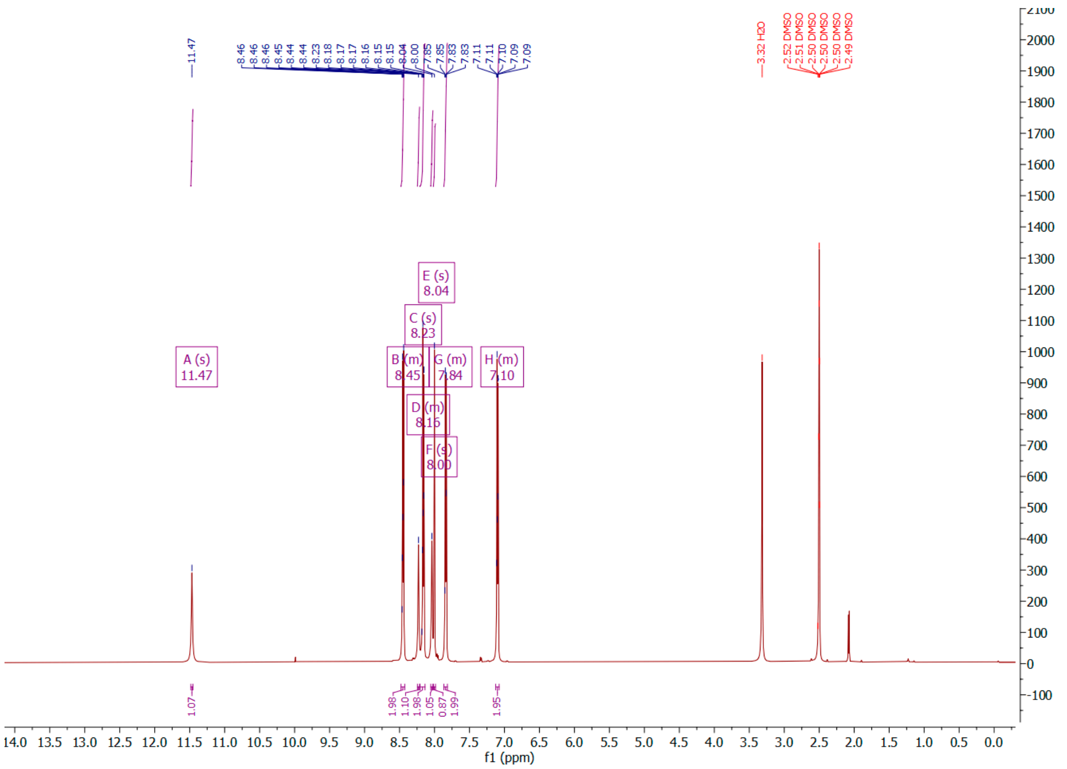

Figure S6. ^{1}H NMR spectrum of **TCBS2**, (*E*)-4-((2-carbamothioylhydrazineylidene)methyl)phenyl 4-nitrobenzenesulfonate (600 MHz, DMSO d-6).

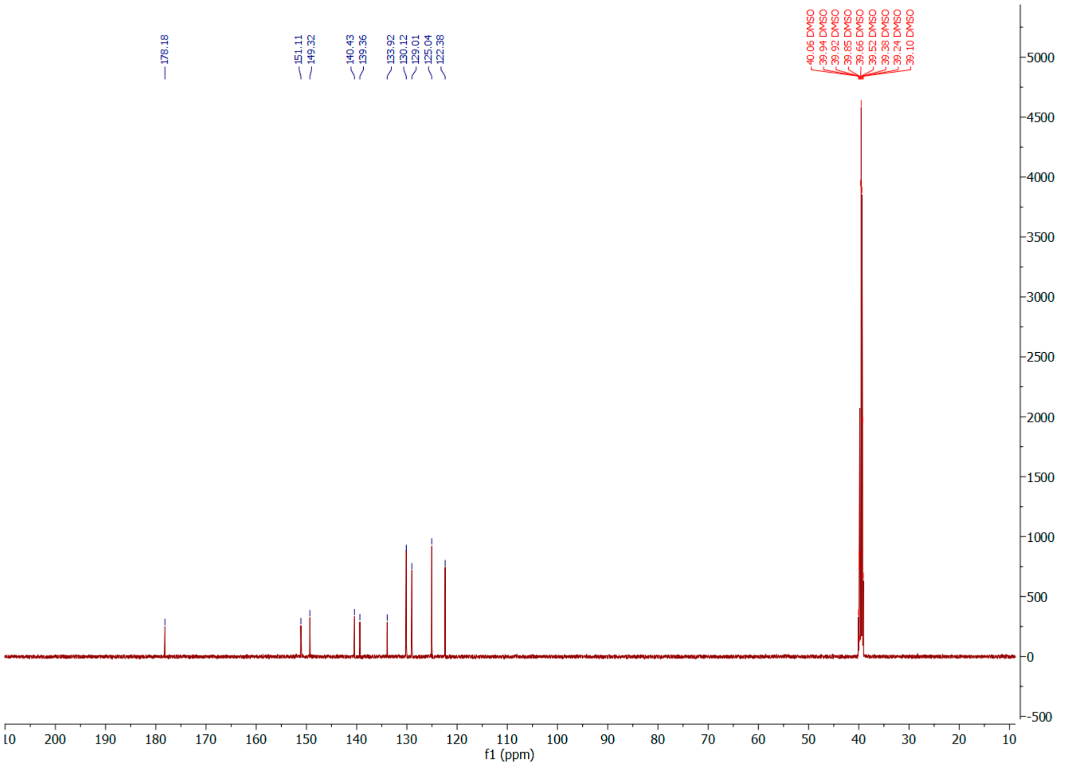

Figure S7. ^{13}C NMR spectrum of **TCBS2**, (*E*)-4-((2-carbamothioylhydrazineylidene)methyl)phenyl 4-nitrobenzenesulfonate (151 MHz, DMSO d-6).

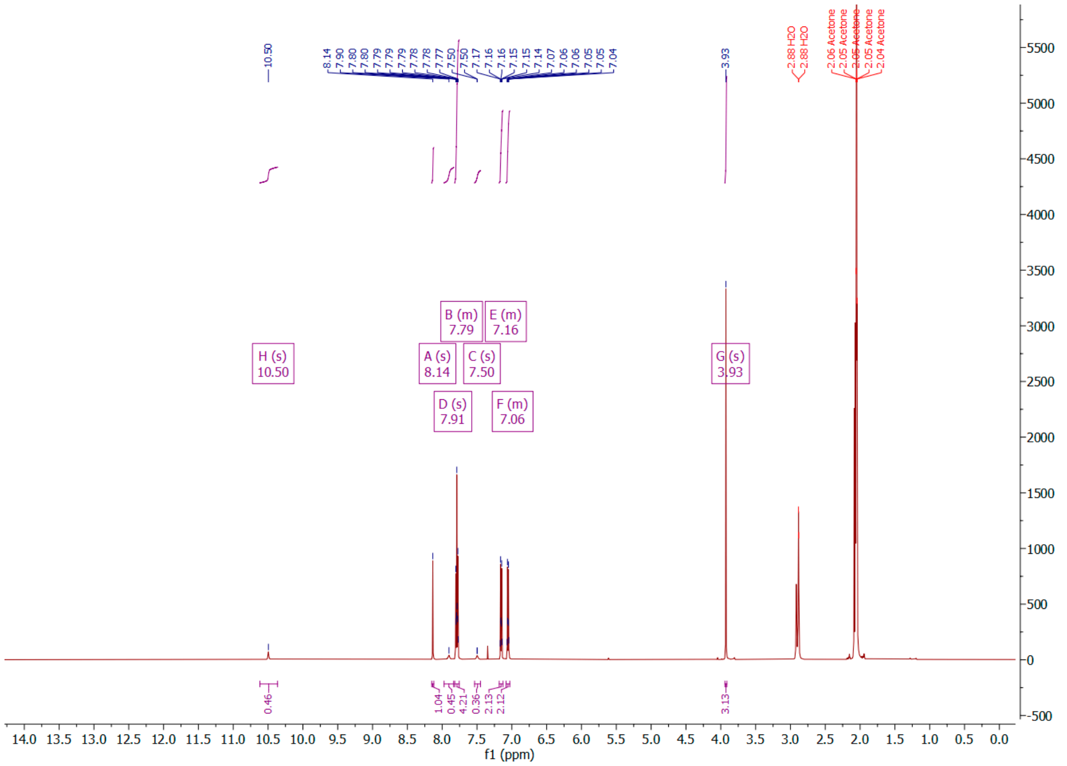

Figure S8. ^{1}H NMR spectrum of **TCBS3**, (*E*)-4-((2-carbamothioylhydrazineylidene)methyl)phenyl 4-methoxybenzenesulfonate (600 MHz, Acetone d-6).

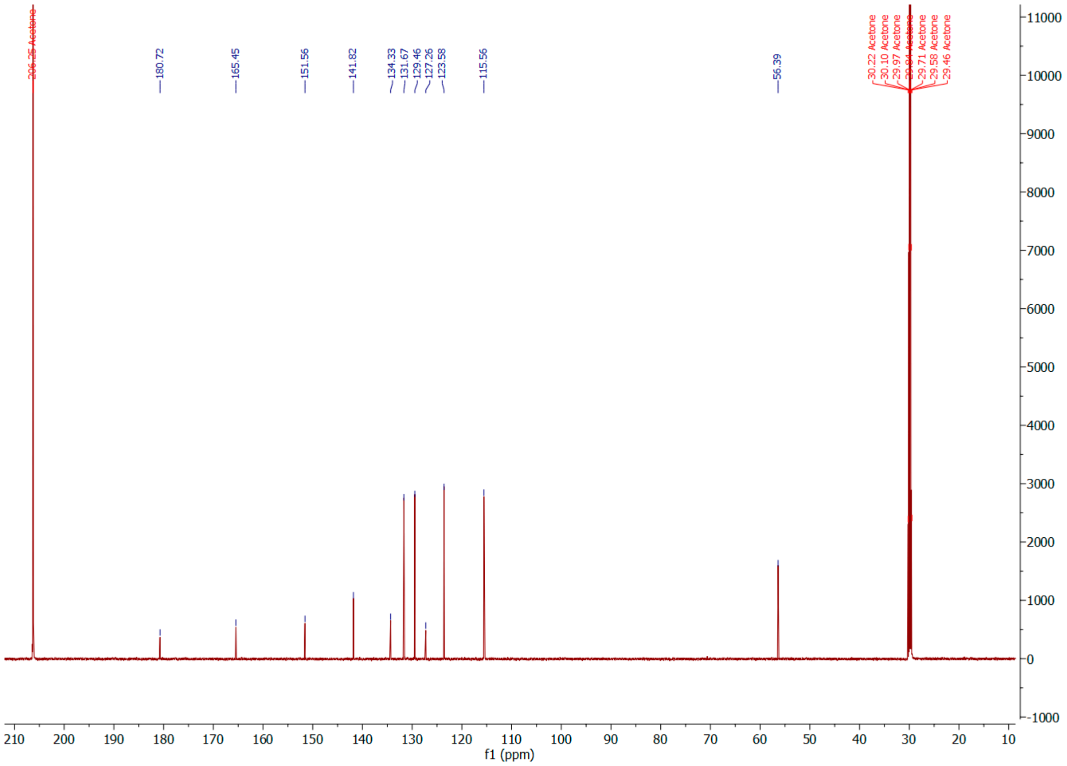

Figure S9. ^{13}C NMR spectrum of **TCBS3**, (*E*)-4-((2-carbamothioylhydrazineylidene)methyl)phenyl 4-methoxybenzenesulfonate (151 MHz, Acetone d-6).

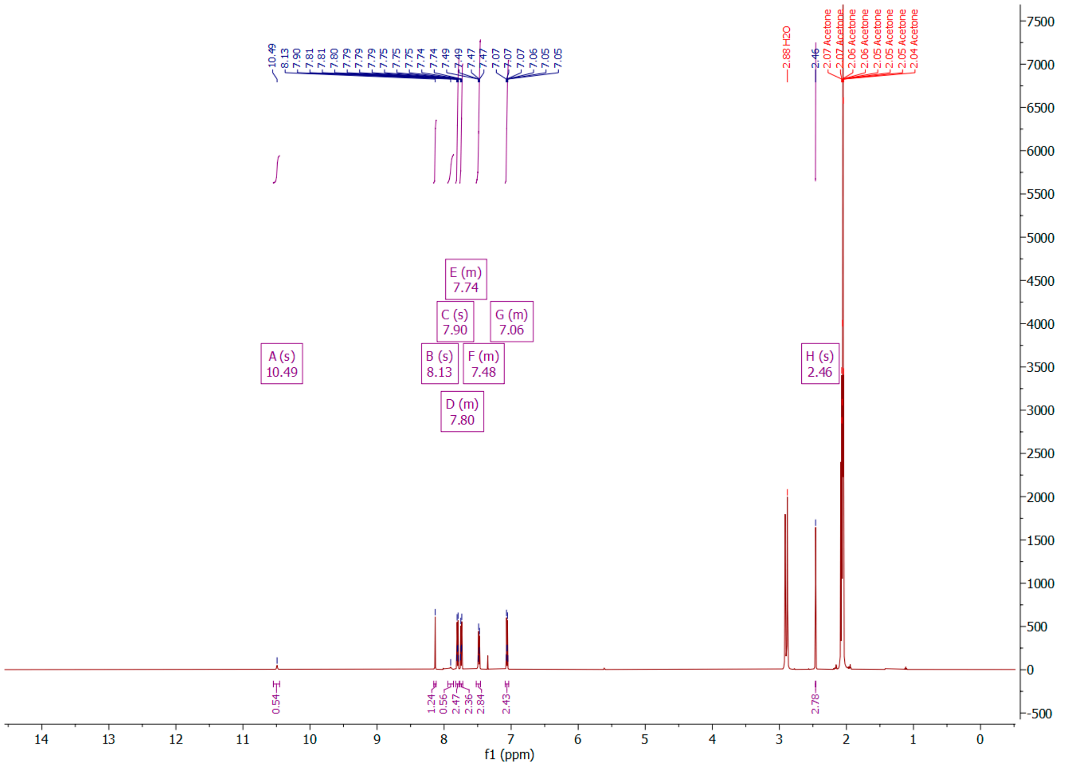

Figure S10. ^{1}H NMR spectrum of **TCBS4**, (*E*)-4-((2-carbamothioylhydrazineylidene)methyl)phenyl 4-methylbenzenesulfonate (600 MHz, Acetone d-6).

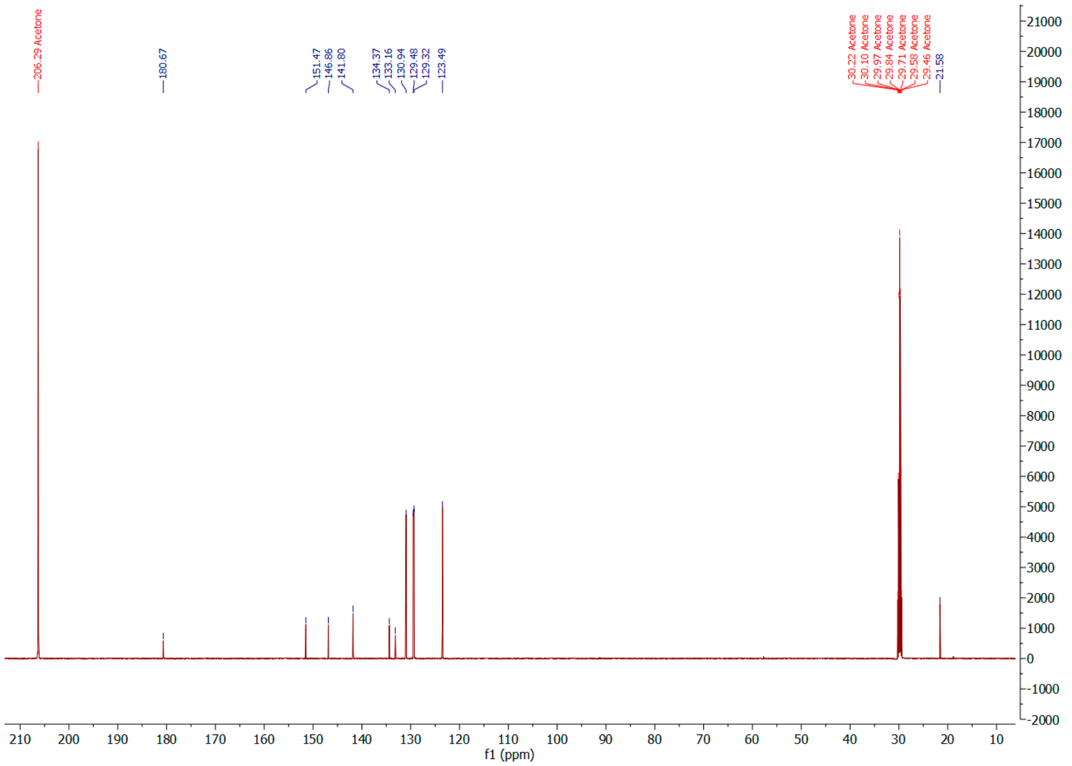

Figure S11. ^{13}C NMR spectrum of **TCBS4**, (*E*)-4-((2-carbamothioylhydrazineylidene)methyl)phenyl 4-methylbenzenesulfonate (151 MHz, Acetone d-6).

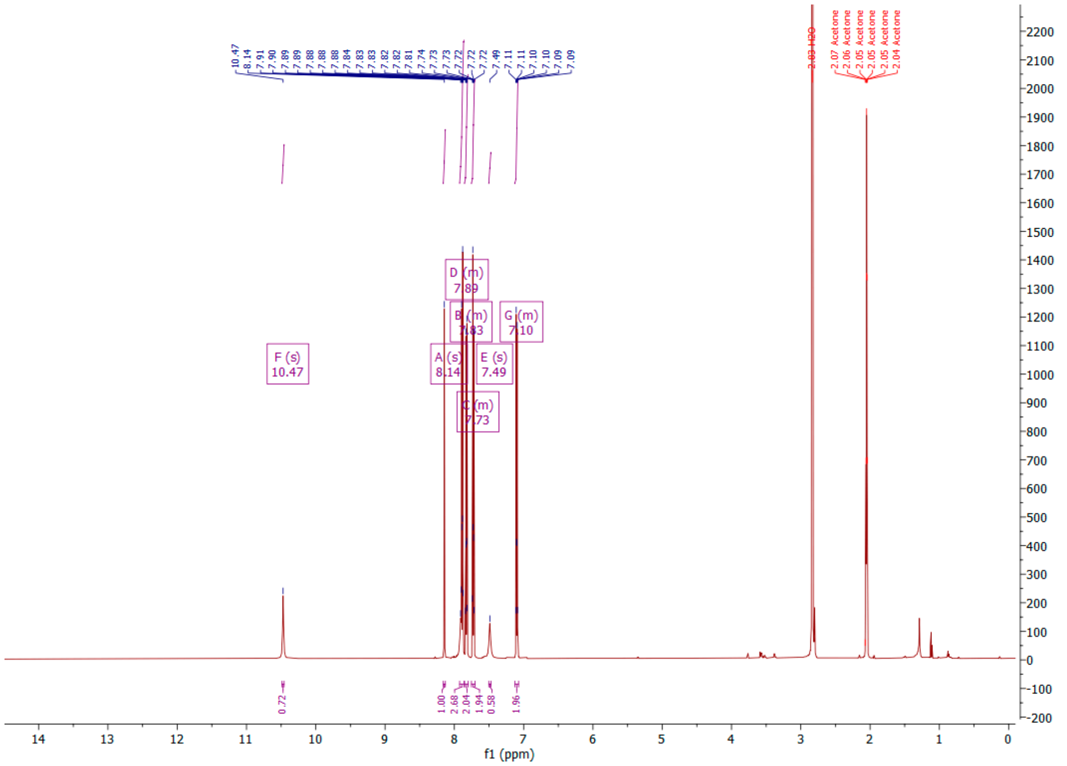

Figure S12. ^{1}H NMR spectrum of **TCBS5**, (*E*)-4-((2-carbamothioylhydrazineylidene)methyl)phenyl 4-chlorobenzenesulfonate (600 MHz, Acetone d-6).

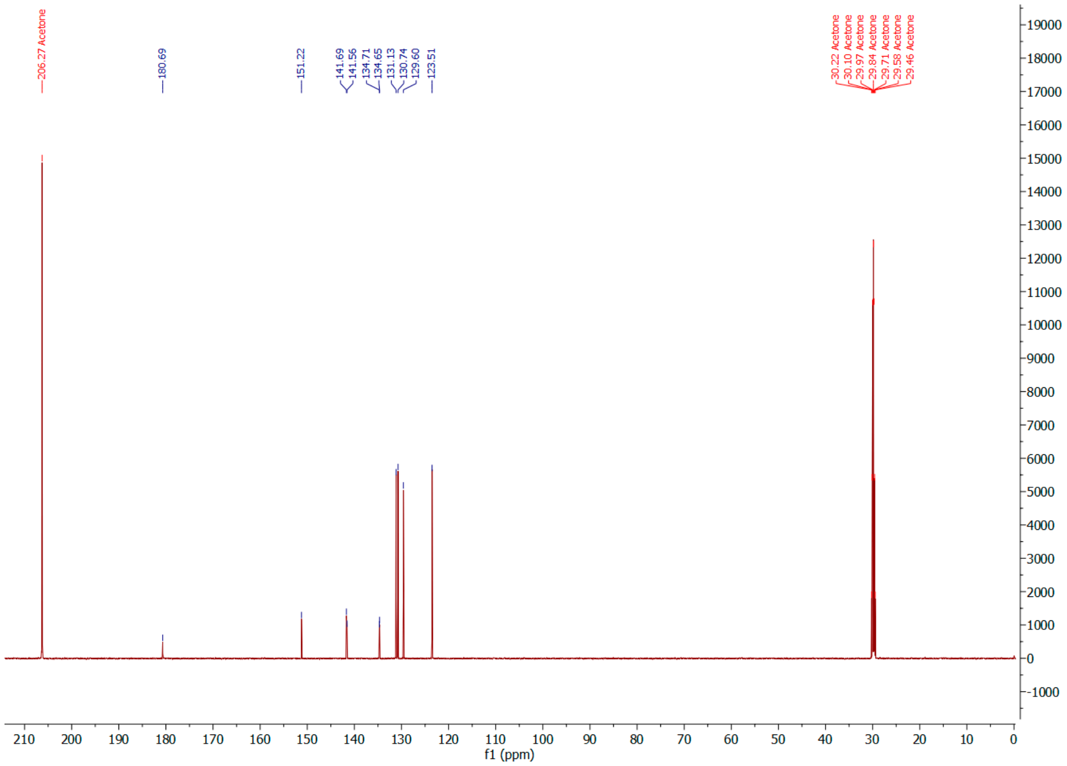

Figure S13. ^{13}C NMR spectrum of **TCBS5**, (*E*)-4-((2-carbamothioylhydrazineylidene)methyl)phenyl 4-chlorobenzenesulfonate (151 MHz, Acetone d-6).

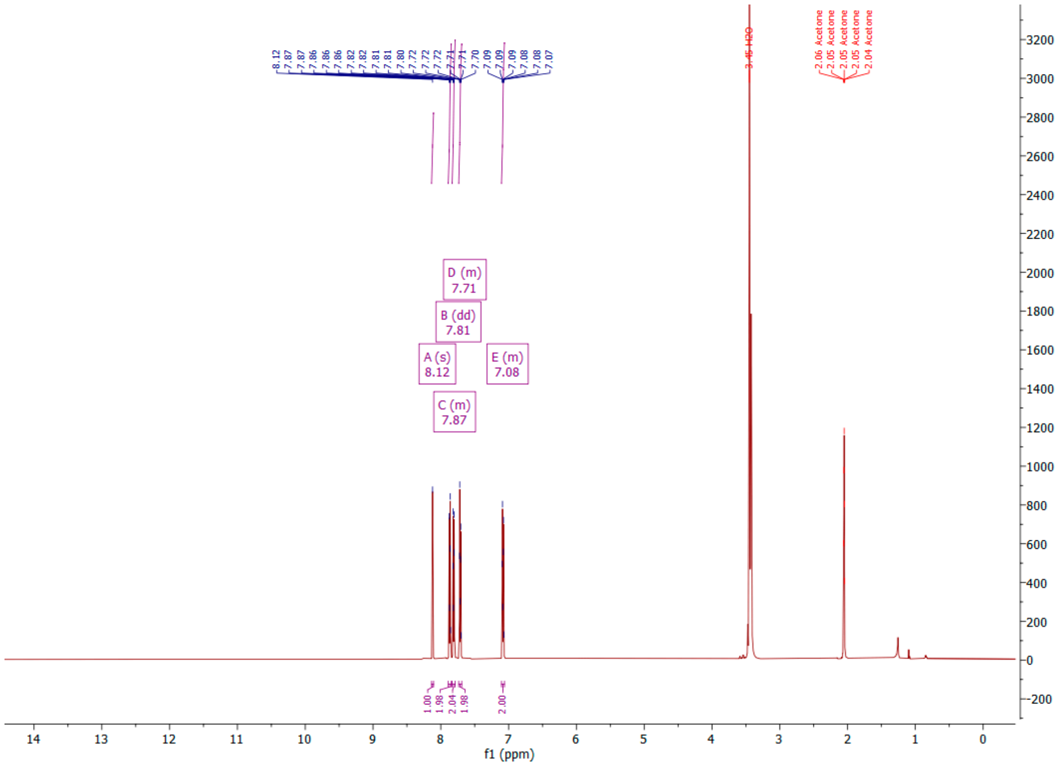

Figure S14. ^{1}H NMR spectrum of **TCBS5**, (*E*)-4-((2-carbamothioylhydrazineylidene)methyl)phenyl 4-chlorobenzenesulfonate after addition of D_2O (600 MHz, Acetone d-6).

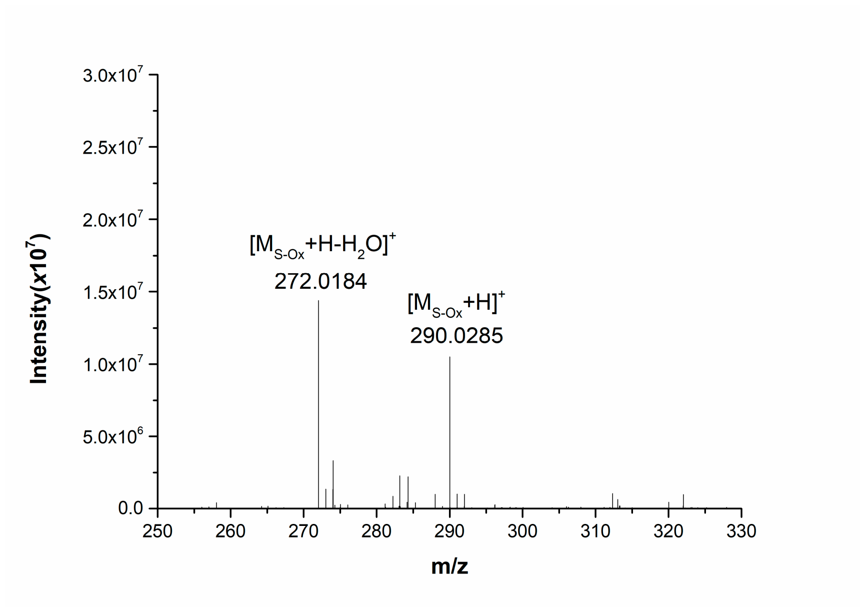

Figure S15. High-Resolution ESI mass spectrum (positive mode) of compound **TCMS1**.

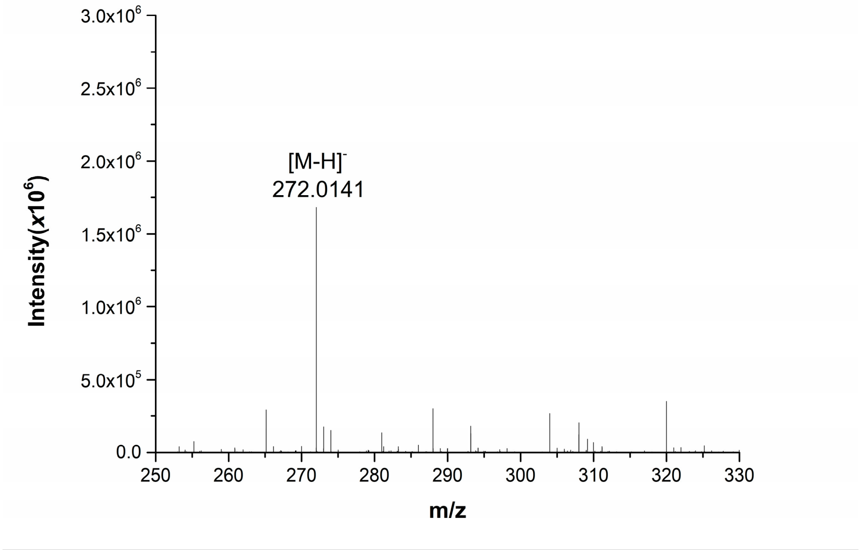

Figure S16. High-Resolution ESI mass spectrum (negative mode) of compound **TCMS1**.

2. Structural characterization

Table S1 Crystal data and structure refinement parameters for **TCSB1**.

CCDC deposition number	2374503
Empirical formula	$C_{14}H_{13}N_3O_3S_2$
Formula weight	335.39
Temperature/K	100(2)
Crystal system	triclinic
Space group	$P\bar{1}$
a/Å	8.0500(4)
b/Å	11.3040(5)
c/Å	17.7084(9)
α/°	85.951(2)
β/°	77.866(2)
γ/°	76.637(2)
Volume/Å^3	1532.35(13)
Z	4
ρ_{calc}g/cm^3	1.454
μ/mm^{-1}	0.363
Crystal size/mm^3	0.56 × 0.06 × 0.02
Radiation	Mo$K\alpha$ (λ = 0.71073)
2θ range for data collection/°	3.704 to 56.622
Reflections collected	67045
Independent reflections	7620 [R_{int} = 0.0489, R_{sigma} = 0.0237]
Data/restraints/parameters	7620/0/415
GooF on F^2	1.031

Final R indexes [I ≥ 2σ (I)]	R_1 = 0.0292, wR_2 = 0.0728
Final R indexes [all data]	R_1 = 0.0357, wR_2 = 0.0777
Largest diff. peak/hole / e Å^{-3}	0.37/−0.47

Table S2 Bond lengths (Å) for compound **TCSB1**.

S4–O4	1.6104(10)	C6–C5	1.3772(19)
S4–O5	1.4254(10)	C4–C3	1.3924(19)
S4–O6	1.4241(10)	C4–C5	1.3905(18)
S4–C23	1.7518(14)	C23–C28	1.3932(19)
S2–O1	1.6133(10)	C23–C24	1.3907(19)
S2–O2	1.4266(11)	C16–C17	1.4623(18)
S2–O3	1.4232(10)	C19–C20	1.3889(19)
S2–C9	1.7489(14)	C19–C18	1.3871(18)
S3–C15	1.6994(13)	C21–C20	1.3833(19)
S1–C1	1.6954(14)	C21–C22	1.3896(19)
O4–C20	1.4096(15)	C17–C18	1.4010(18)
O1–C6	1.4104(15)	C17–C22	1.3966(19)
N6–N5	1.3783(15)	C3–C2	1.4613(18)
N6–C16	1.2806(18)	C3–C8	1.4021(19)
N5–C15	1.3430(17)	C7–C8	1.3841(19)
N4–C15	1.3240(17)	C14–C13	1.389(2)
N2–N3	1.3767(15)	C28–C27	1.383(2)
N2–C1	1.3432(17)	C12–C13	1.388(2)
N3–C2	1.2823(18)	C12–C11	1.390(2)
N1–C1	1.3298(18)	C24–C25	1.388(2)
C9–C14	1.3869(18)	C10–C11	1.384(2)
C9–C10	1.3937(18)	C26–C27	1.391(2)
C6–C7	1.3913(19)	C26–C25	1.385(2)

Table S3 Bond angles (°) for compound **TCSB1**.

O4–S4–C23	102.92(6)	N6–C16–C17	121.71(12)
O5–S4–O4	108.50(6)	C18–C19–C20	118.87(12)
O5–S4–C23	110.13(6)	C20–C21–C22	118.36(13)
O6–S4–O4	102.61(6)	C18–C17–C16	121.80(12)
O6–S4–O5	121.01(6)	C22–C17–C16	118.80(12)
O6–S4–C23	109.91(6)	C22–C17–C18	119.40(12)
O1–S2–C9	101.36(6)	N2–C1–S1	119.09(10)
O2–S2–O1	108.24(6)	N1–C1–S1	122.97(11)
O2–S2–C9	109.79(6)	N1–C1–N2	117.92(13)
O3–S2–O1	103.02(6)	C4–C3–C2	117.88(12)
O3–S2–O2	120.84(7)	C4–C3–C8	119.57(12)
O3–S2–C9	111.50(6)	C8–C3–C2	122.52(12)

C20–O4–S4	119.47(8)	C19–C20–O4	119.98(12)
C6–O1–S2	117.85(8)	C21–C20–O4	117.60(12)
C16–N6–N5	114.79(11)	C21–C20–C19	122.25(12)
C15–N5–N6	119.86(11)	C8–C7–C6	119.00(13)
C1–N2–N3	120.05(12)	C19–C18–C17	120.23(12)
C2–N3–N2	114.12(11)	C21–C22–C17	120.85(12)
C14–C9–S2	118.74(10)	C6–C5–C4	118.67(12)
C14–C9–C10	122.14(13)	C9–C14–C13	118.65(13)
C10–C9–S2	118.78(10)	N3–C2–C3	122.41(12)
C7–C6–O1	119.35(12)	C7–C8–C3	120.06(13)
C5–C6–O1	118.45(12)	C27–C28–C23	118.70(13)
C5–C6–C7	122.03(12)	C13–C12–C11	120.81(13)
N5–C15–S3	119.06(10)	C25–C24–C23	118.17(13)
N4–C15–S3	122.50(10)	C11–C10–C9	118.45(13)
N4–C15–N5	118.39(12)	C12–C13–C14	119.86(13)
C5–C4–C3	120.65(13)	C25–C26–C27	120.40(14)
C28–C23–S4	118.03(10)	C28–C27–C26	120.09(14)
C24–C23–S4	119.92(11)	C10–C11–C12	120.07(13)
C24–C23–C28	122.03(13)	C26–C25–C24	120.59(13)

Table S4 Comparison between the mean bond distances (Å) calculated for the thiosemicarbazone moieties in the two units in **TCBS1** and the mean values retrieved from the CSD (version 5.45 updated Mar 2024).

ConQuest Query	Parameter	Mean values based on CSD search: 5146 hits	Mean values for TCBS1
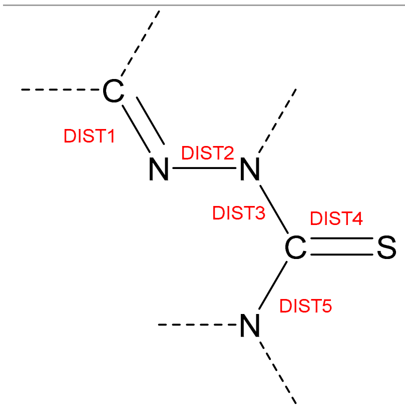	DIST1	1.288(15)	1.281
	DIST2	1.378(17)	1.378
	DIST3	1.351(21)	1.343
	DIST4	1.689(24)	1.697
	DIST5	1.336(25)	1.327

a)

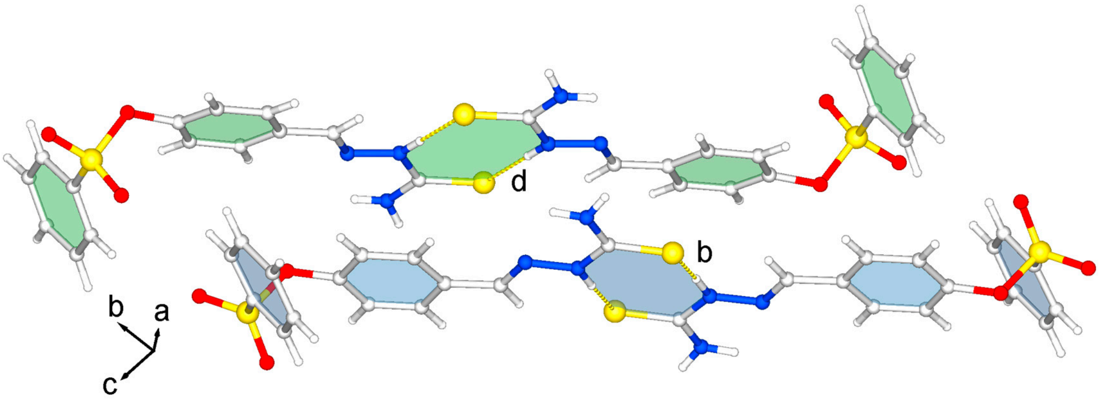

b) **c)**

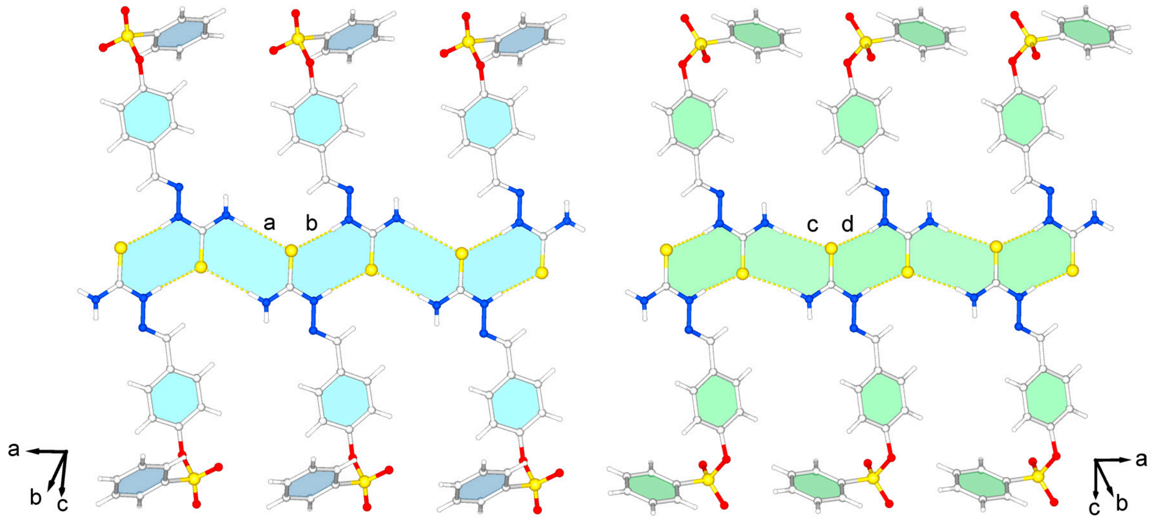

Figure S17. Hydrogen bonding network found in the crystal structure of **TCBS1**: a) partial view on the relative orientation between units A and B. b) and c) show the infinite hydrogen-bonded network for units A and B, respectively. Interactions are labelled according to **Table S5**.

Table S5. Intermolecular hydrogen bonding interactions of **TCBS1**.

		N–H···S	$d_{\mathrm{N–H}}$ (Å)	$d_{\mathrm{H···S}}$ (Å)	$d_{\mathrm{N···F}}$ (Å)	$\alpha_{\mathrm{N–H···S}}$ (°)
Unit A	*a*	N1^{i}–H1B^{i}···S1	0.83(2)	2.67(2)	3.476(2)	164(2)
	b	N2^{ii}–H2^{ii}···S1	0.88(2)	2.54(2)	3.375(2)	160(2)
Unit B	*c*	N4^{iii}–H4b^{iii}···S3	0.87(2)	2.54(2)	3.374(2)	161(2)
	d	N5^{iv}–H5^{iv}···S3	0.85(2)	2.46(2)	3.293(2)	167(2)

Symmetry codes: ^{i} = 3−*x*, −*y*, 1−*z*; ^{ii} = 2−*x*, −*y*, 1−*z*; ^{iii} = 1−*x*, 1−*y*, 1−*z*; ^{iv} = 2−*x*, 1−*y*, 1−*z*.

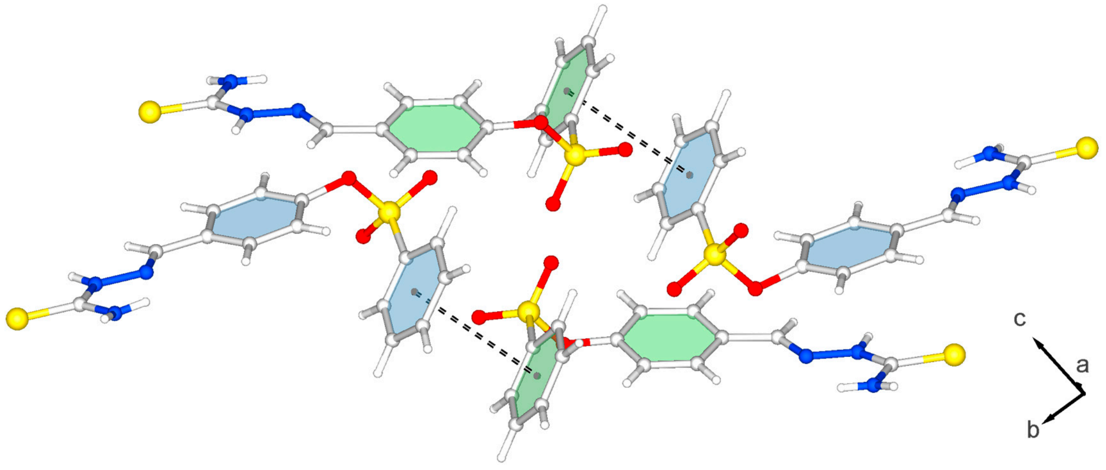

Figure S18. Intermolecular π-π stacking interactions between tosyl groups in the crystal structure of **TCBS1**. Intercentroid distance: 3.90 Å; shift distance: 1.46 Å; plane to plane angle: 9°.

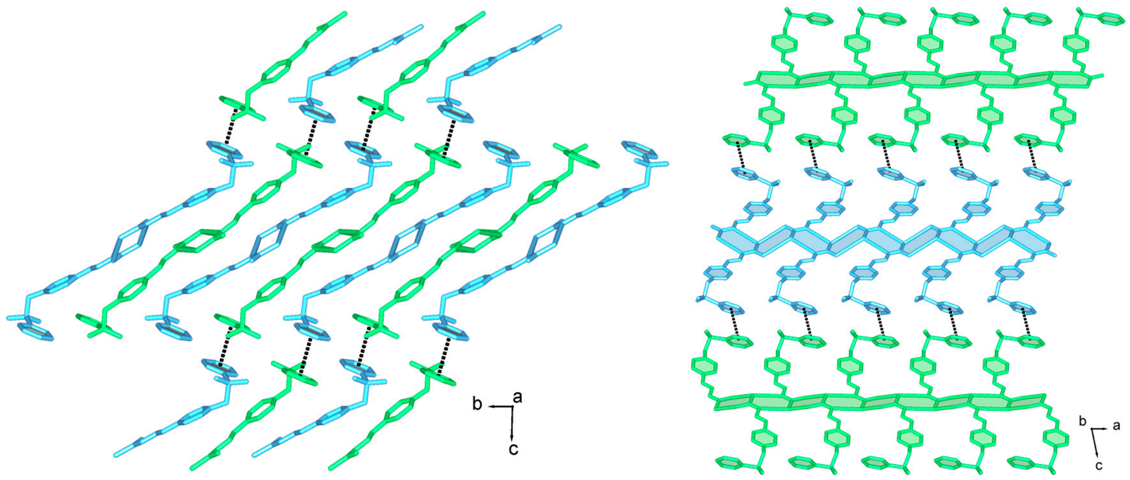

Figure S19. Partial view of the packing diagrams of **TCBS1** along the *a*- (left) and *b*-axis (right). Units A and B are depicted in light blue and green, respectively.

3. Copper Chelation studies

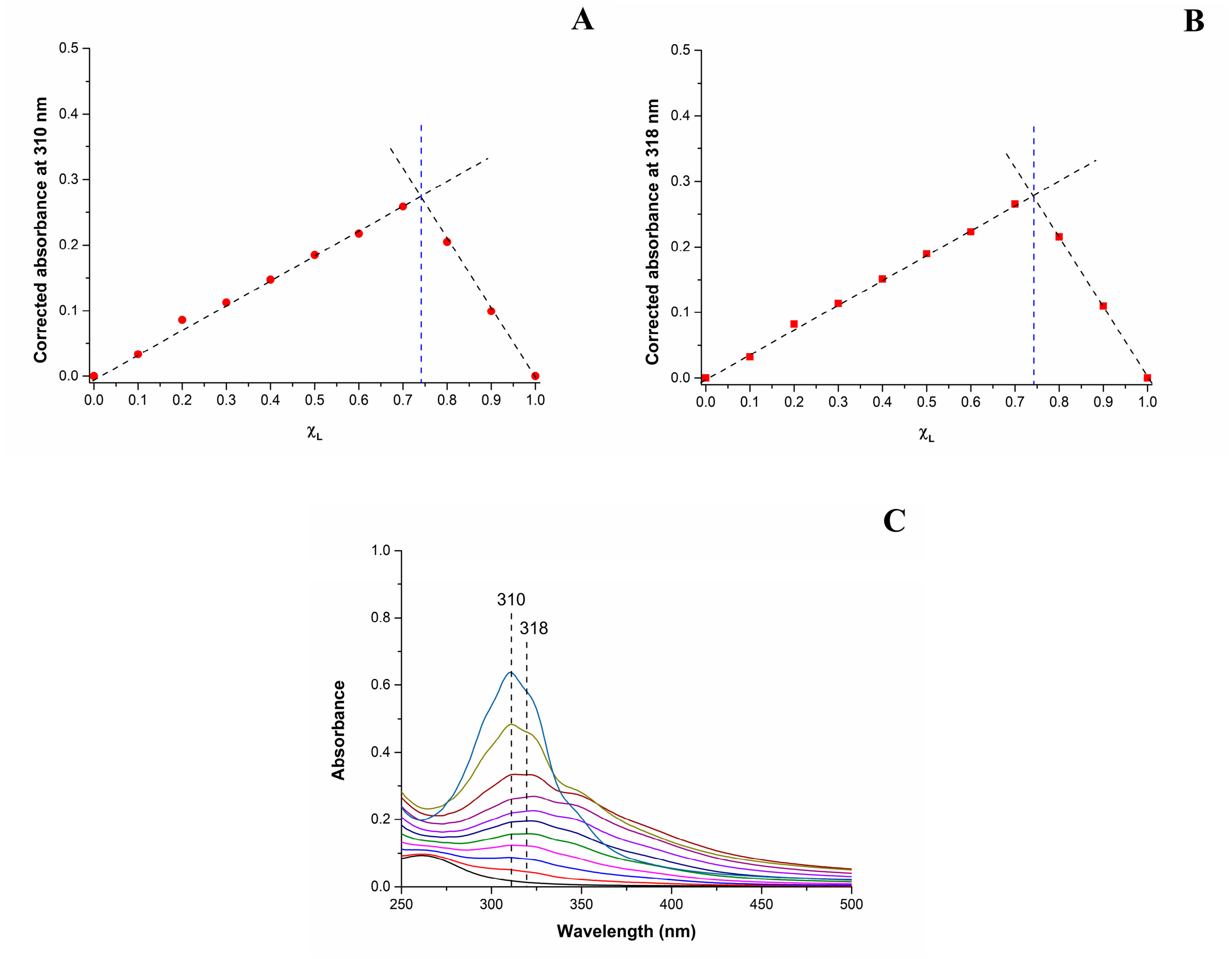

Figure S20. Job's plot of Cu^{2+} and **TCBS4** at 310 nm (**A**) and 318 (**B**); (**C**) Absorption spectra collected by varying $Cu^{2+}$and **TCBS4** molar ratios in in PB 0.05 M, pH 6.8, 25 °C, 1 cm optical path length.

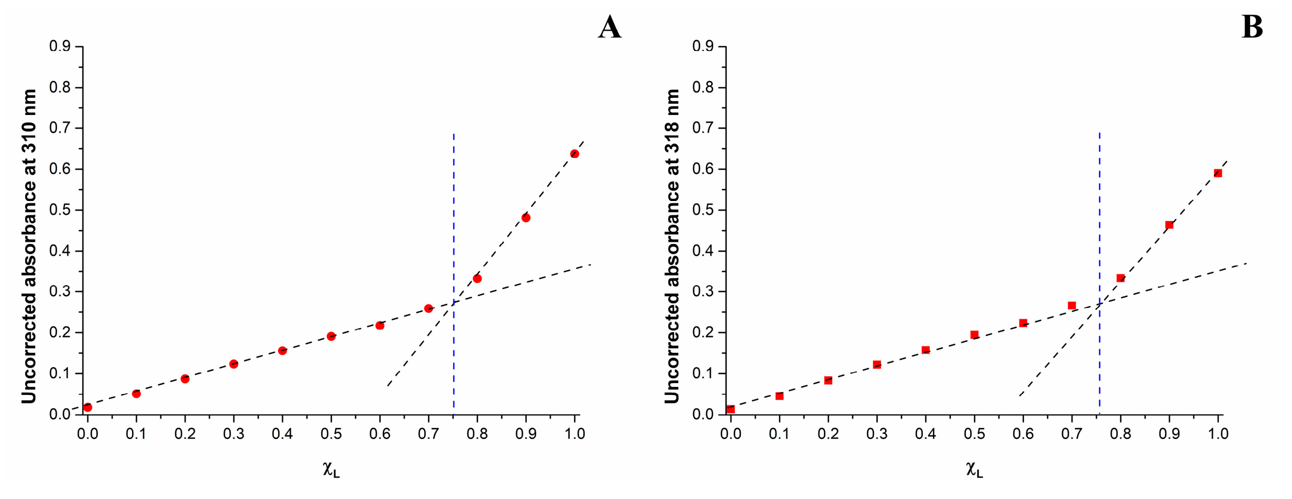

Figure S21. Uncorrected absorbance data at 310 nm (**A**) and 318 nm (**B**) recorded by varying $Cu^{2+}$and **TCBS4** molar ratios.

4. Molecular Docking

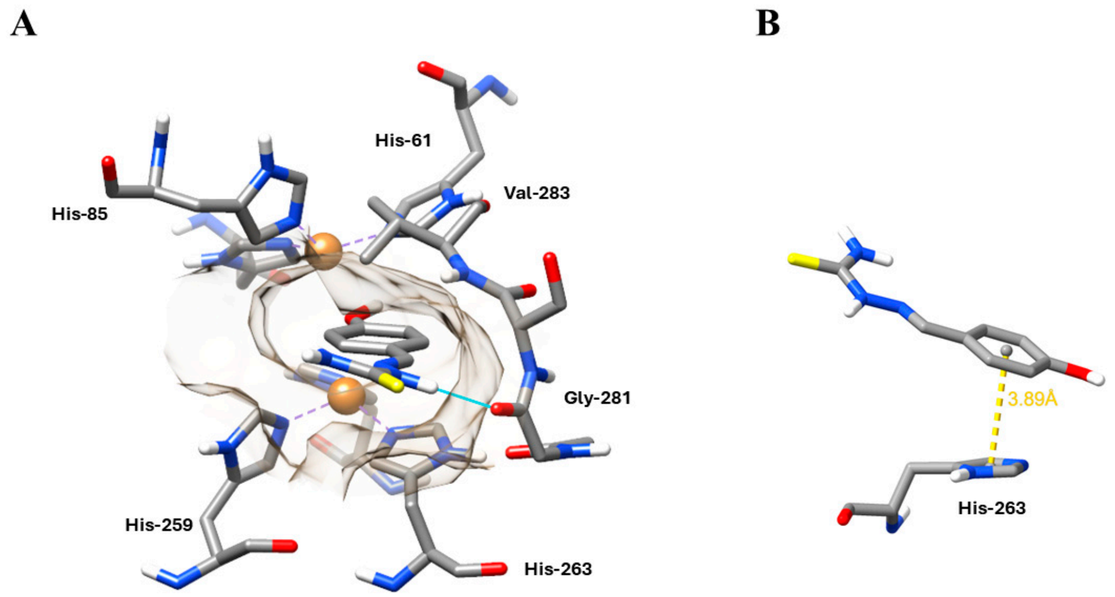

Figure S22. Docked pose of **TC1** in the MTa site and intermolecular interactions with the surrounding residues. Hydrogen bonds are represented using light blue solid lines, while metal coordinating bonds are shown as violet dashed ones (**A**). π-π interaction between the docked pose of **TC1** and the Hys-263 residue.

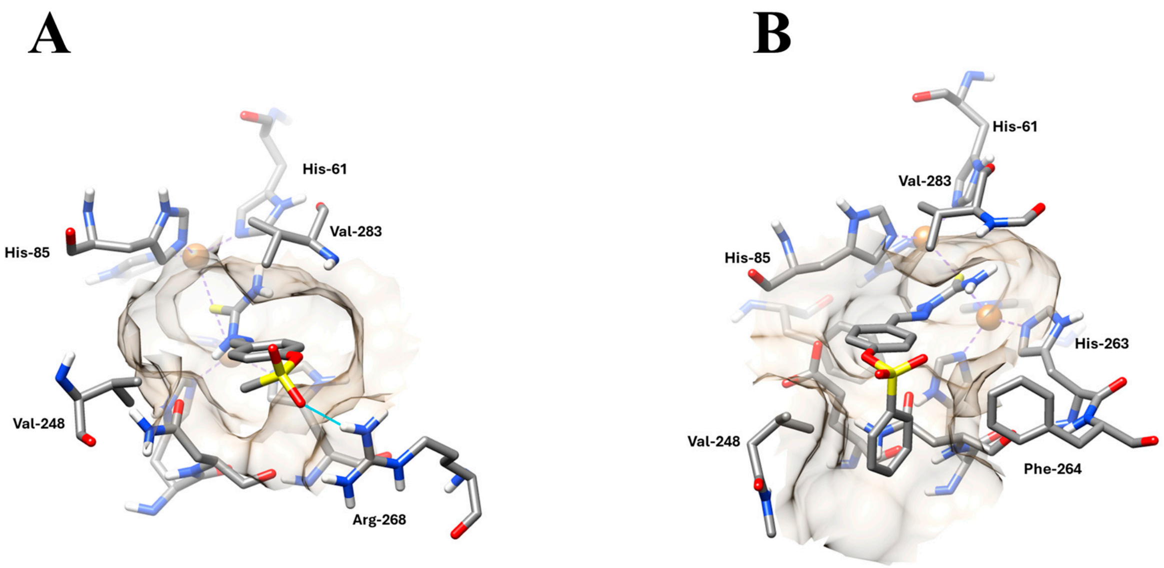

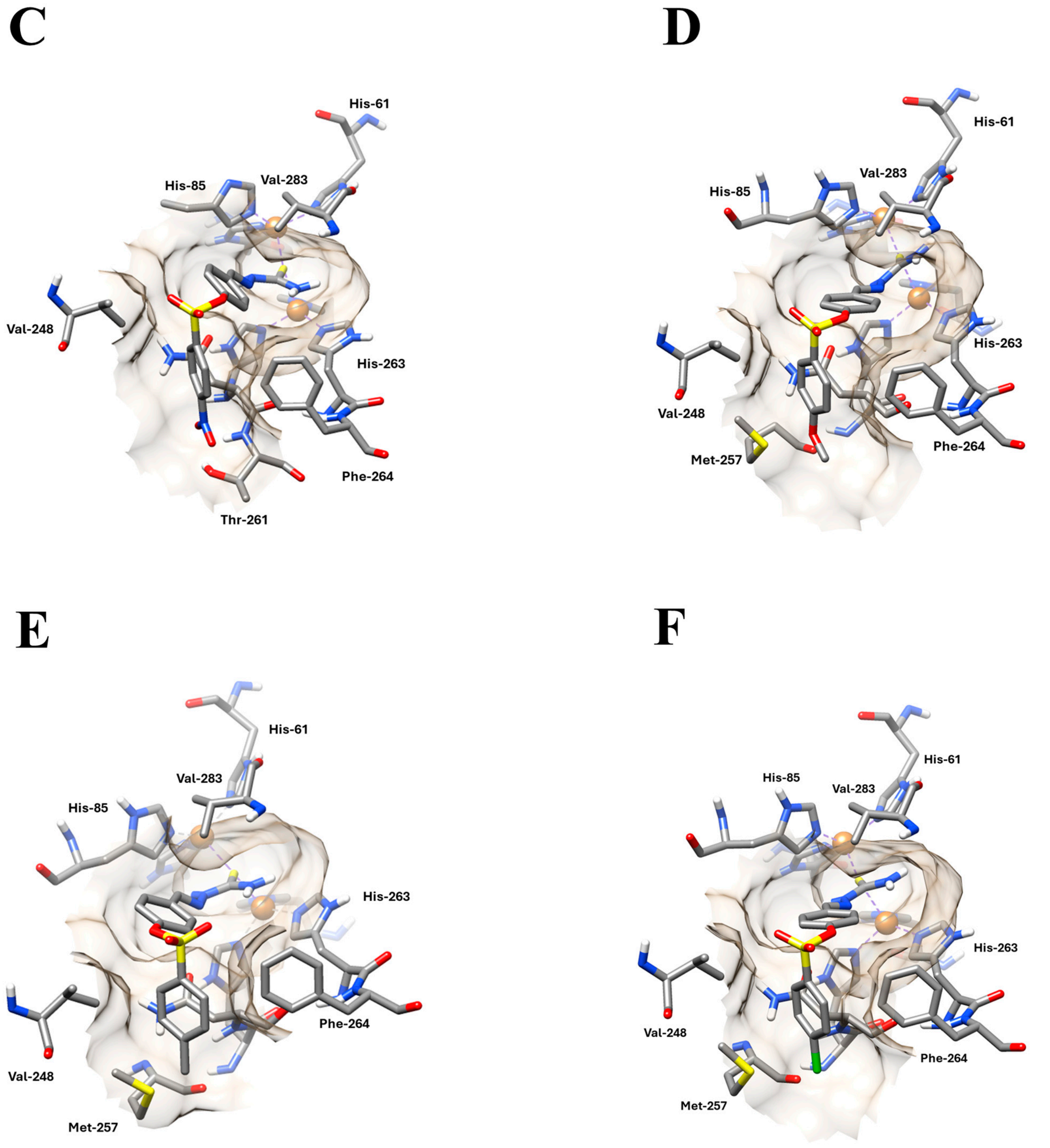

Figure S23. Docked poses of **TCMS1 (A)**, **TCBS1-5 (B-F)** in the MTa site and intermolecular interactions with the surrounding residues. Hydrogen bonds are represented using light blue solid lines, while metal coordinating bonds are shown as violet dashed ones.

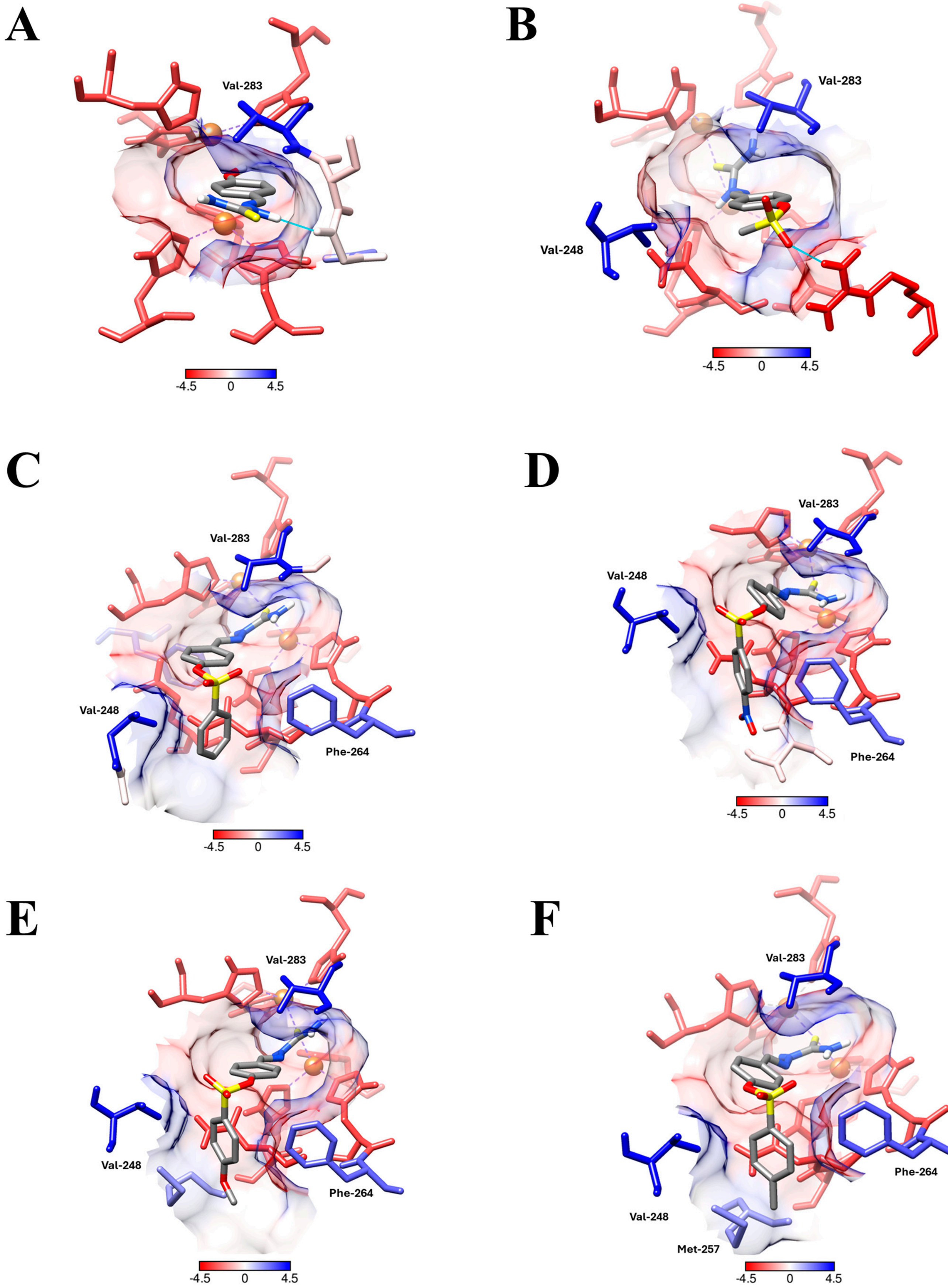
A
Val-283
-4.5
0
4.5
B
Val-283
Val-248
-4.5
0
4.5
C
Val-283
Val-248
Phe-264
-4.5
0
4.5
D
Val-283
Val-248
Phe-264
-4.5
0
4.5
E
Val-283
Val-248
Phe-264
-4.5
0
4.5
F
Val-283
Val-248
Met-257
Phe-264
-4.5
0
4.5

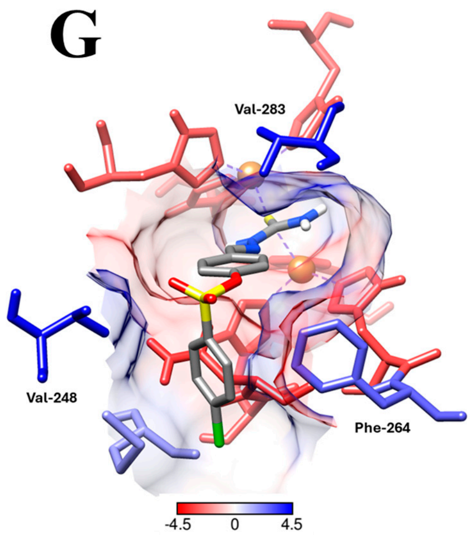

Figure S24. Hydrophobic interaction analysis between the docked poses of **TC1** (**A**), **TCMS1** (**B**), **TCBS1-5** (**C-G**) and the surrounding residues of mushroom tyrosinase (MTa site). The aminoacidic residues are coloured according to the Kyte-Doolittle hydrophobicity scale values.

Table S6. Scores, molecular interactions and distances between the highest-rated docked poses of compounds **TC1**, **TCMS1**, **TCBS1-5** and the surrounding residues of mushroom tyrosinase (MTa).

Compound	Docking Score	Ligand's functional groups	Receptor's residues/ cofactors	Interaction type	Distance (Å)
**TC1**	56.86	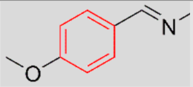	His-263	π- π stacked	3.89 (ring centroids)
		-NH	Gly-281	hydrogen bond	1.96 (D-H--A)
		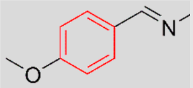	Val-283	hydrophobic	3.42 (CH_3-C_{arom})*
**TCMS1**	31.26	C=S	Cu-400	metal-acceptor	2.45
		C=S	Cu-401	metal-acceptor	2.40
		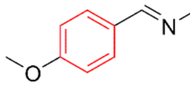	Val-283	hydrophobic	3.42 (CH_3-C_{arom})*
**TCBS1**	40.87	C=S	Cu-400	metal-acceptor	2.33
		C=S	Cu-401	metal-acceptor	2.42
		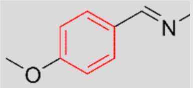	Val-283	hydrophobic	3.89 (CH_3-C_{arom})*
		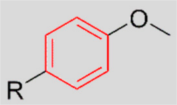	Val-248/ Phe-264	hydrophobic	3.70 (CH_3--C_{arom})* 3.68 (C_{arom}--C_{arom})*
**TCBS2**	43.61	C=S	Cu-400	metal-acceptor	2.44
		C=S	Cu-401	metal-acceptor	2.39
		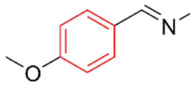	Val-283	hydrophobic	3.59 (CH_3-C_{arom})*
		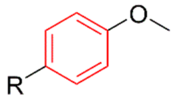	Val-248/ Phe-264	hydrophobic	3.08 (CH_3--C_{arom})* 3.45 (C_{arom}--C_{arom})*
**TCBS3**	41.31	C=S	Cu-400	metal-acceptor	2.22
		C=S	Cu-401	metal-acceptor	2.35
		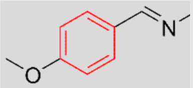	Val-283	hydrophobic	3.42 (CH_3-C_{arom})*
				hydrophobic	

		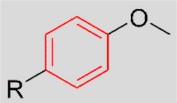	Val-248/<br>Phe-264		3.66 (CH_3--C_{arom})*<br>3.35 (C_{arom}--C_{arom})*
**TCBS4**	43.79	C=S	Cu-400	metal-acceptor	2.53
		C=S	Cu-401	metal-acceptor	2.20
		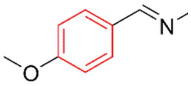	Val-283	hydrophobic	3.55 (CH_3-C_{arom})*
		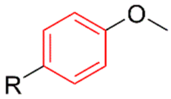	Val-248/<br>Phe-264	hydrophobic	3.08 (CH_3--C_{arom})*<br>3.46 (C_{arom}--C_{arom})*
**TCBS5**	41.33	C=S	Cu-400	metal-acceptor	2.11
		C=S	Cu-401	metal-acceptor	2.50
		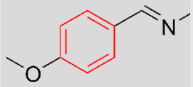	Val-283	hydrophobic	3.53 (CH_3-C_{arom})*
		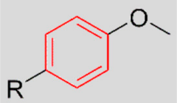	Val-248/<br>Phe-264	hydrophobic	3.55 (CH_3--C_{arom})*<br>3.34 (C_{arom}--C_{arom})*

*The values reported are referred to the closest carbon distances between the ligand's aryl moieties and the residues' alkyl/phenyl groups.
